# Dietary Patterns Associated With Anti-inflammatory Effects: An Umbrella Review of Systematic Reviews and Meta-analyses

**DOI:** 10.1093/nutrit/nuaf104

**Published:** 2025-07-14

**Authors:** Gynette L Reyneke, Kelly Lambert, Eleanor J Beck

**Affiliations:** School of Health Sciences, University of New South Wales, Sydney, New South Wales 2052, Australia; School of Medical, Indigenous, and Health Sciences, University of Wollongong, Wollongong, New South Wales 2522, Australia; School of Health Sciences, University of New South Wales, Sydney, New South Wales 2052, Australia; School of Medical, Indigenous, and Health Sciences, University of Wollongong, Wollongong, New South Wales 2522, Australia

**Keywords:** dietary pattern, anti-inflammatory, umbrella review, Mediterranean diet

## Abstract

**Context:**

Noncommunicable diseases significantly impact global health, and chronic inflammation is a common pathological feature of these conditions. The relationship between chronic inflammation and dietary intake is increasingly evident, as emerging research elucidates the inflammation-modulating effects of diet.

**Objective:**

This umbrella review aimed to systematically collect, summarize, and assess current evidence on the relationship between dietary patterns and inflammatory biomarkers.

**Data Sources:**

The CINAHL, Cochrane Library, PubMed, Scopus, and Web of Science databases were searched from 1990 through March 19, 2025.

**Data Extraction:**

Systematic reviews and meta-analyses of intervention trials and observational studies that assessed the effects or associations of dietary patterns on chronic inflammatory markers were selected. Data extraction, methodological quality assessment, and evaluation of the strength of evidence were independently conducted by 2 authors.

**Data Analysis:**

Thirty reviews representing 225 eligible primary studies were included. Fifteen dietary patterns were assessed against a range of inflammatory marker outcomes, reported in 60 unique meta-analyses and 61 narrative syntheses. The findings indicate significant effects and overall beneficial association between the Mediterranean diet and the levels of inflammatory markers C-reactive protein (CRP), interleukin-6, and adiponectin, with the certainty of evidence (CoE) ranging from high to low. Additionally, a significant inverse association was identified between a vegetarian diet and CRP levels, with low to very low CoE. The findings for other dietary patterns assessed were inconclusive or limited due to the paucity of studies.

**Conclusion:**

The Mediterranean and vegetarian dietary patterns may ameliorate low-grade inflammation in adult populations with at least one chronic condition. Further studies are needed to evaluate the potential inflammation-modulating effects of other dietary patterns, considering the significant heterogeneity of comparator diets.

**Systematic Review Registration:**

PROSPERO no. CRD42023472469.

## INTRODUCTION

Noncommunicable diseases (NCDs) encompass a group of chronic conditions that profoundly impact global morbidity and mortality, while imposing substantial socioeconomic consequences.^1–3^ The etiology of NCDs typically involves a complex interplay of genetic, environmental, and lifestyle factors.^1^^,^^3^ Diet is considered a dominant modifiable risk factor for the development and progression of various chronic conditions, including cardiovascular disease (CVD), type 2 diabetes (T2D), certain cancers, and neurodegenerative diseases.^3^^,^^4^ Although diet may not directly prevent the onset of all NCDs, for instance, autoimmune conditions, nutrition is foundational in the management of conditions such as type 1 diabetes (T1D).^3^^,^^5^^,^^6^ Additionally, dietary modification is often used as an adjunctive approach to conventional medical therapy for conditions such as inflammatory bowel diseases (IBDs).^7–9^

Increasingly, emerging evidence indicates that low-grade (chronic) inflammation is a common pathological feature implicated in the development and progression of NCDs.^1^^,^^10^ This persistent state of inflammation, characterized by elevated production of proinflammatory biomarkers such as C-reactive protein (CRP), interleukin-6 (IL-6), tumor necrosis factor alpha (TNF-α), and fibrinogen, disrupts normal physiological processes, leading to tissue damage and dysfunction, and an increased risk of debilitating illnesses.^10^^,^^11^ The relationship between chronic inflammation and dietary patterns (DPs) is becoming increasingly evident, as emerging research elucidates the inflammation-modulating effects of diet.^12^ Plant foods, such as green leafy vegetables, legumes, and whole grains, which are rich in complex carbohydrates, magnesium, fiber, and antioxidants, are associated with lower CRP and IL-6 concentrations.^13–16^

Recently, nutritional research has transitioned from a traditional reductionist approach, examining isolated nutrients and single foods, to a more holistic approach, examining whole DPs in relation to health outcomes. This approach acknowledges the multifaceted interactions between dietary components and their impact on NCD risk.^17^^,^^18^ It emphasizes that the complex interplay between multiple nutrients, foods, and beverages consumed habitually has a greater effect than individual components.^18^ The relationship between inflammation and chronic diseases appears to be inversely correlated with plant-based DPs, which emphasize the consumption of minimally processed plant foods, including vegetables, fruits, whole grains, legumes, nuts, and seeds.^19–21^ These dietary components form the basis of several well-established DPs including the Mediterranean, Dietary Approaches to Stop Hypertension (DASH), and Portfolio diets, all of which are associated with reduced inflammation.^22–24^ For example, the Mediterranean diet is typically characterized by these plant-based foods, ideally locally sourced and seasonal, complemented by moderate fish and poultry consumption, with olive oil as the primary source of dietary fat.^25^^,^^26^ The Mediterranean diet includes moderate consumption of red wine with meals and limited intake of red meat and processed foods.^25^^,^^26^ In contrast, a proinflammatory DP such as that defined as a “Western diet,” typically consists of a high consumption of animal-based and processed foods rich in saturated fat, added sugars, sodium, and refined grains, coupled with a low intake of fiber-rich, nutrient-dense foods.^19^^,^^21^ A proinflammatory DP has been consistently linked to poorer health outcomes and an increased risk of NCDs.^27–29^ The objective of this umbrella review was to systematically collect, summarize, and assess current evidence from systematic reviews and meta-analyses on the relationship between DPs and biomarkers of chronic inflammation. This review incorporated data from both intervention trials, used to determine causality, and observational studies, which offer valuable insights into real-world dietary behaviors and their impact on public health,^30^ to provide a robust summary and critique of the current state of knowledge regarding this widely researched topic.^4^^,^^30^^,^^31^

## METHODS

The current umbrella review was conducted in accordance with the Cochrane Collaboration methodology for Overviews of Reviews,^32^ and followed the reporting guidelines for Preferred Reporting Items for Overviews of Reviews (PRIOR)^33^ (**Table S1**). This review was formulated to answer the research question, “What is the strength of the evidence assessing DPs associated with anti-inflammatory effects?” The protocol for the current umbrella review was registered on the International Prospective Register of Systematic Reviews (PROSPERO) and is available at https://www.crd.york.ac.uk/prospero (ID: CRD42023472469).

### Search Strategy

Peer-reviewed literature published in English was identified by systematically searching scientific databases CINAHL (via EBSCO), Cochrane Library, PubMed, Scopus, and Web of Science from 1990 through March 19, 2025. The search strategy contained both controlled vocabulary (MeSH [Medical Subject Heading] terms) and free text that related to the research question: (Diet OR dietary pattern OR eating pattern OR diet therapy) AND (inflammation OR inflammatory OR anti-inflammation OR anti-inflammatory OR inflammation mediators) AND (systematic review OR systematic literature review OR meta-analysis). The reference lists of eligible articles were manually checked to identify additional relevant studies. The full search strategy is presented in **Table S2**.

### Inclusion and Exclusion Criteria

The eligibility criteria are listed in **Table 1**. Systematic reviews, with or without meta-analyses, were considered eligible for inclusion if they (1) assessed the effect/association between whole DPs and serum concentrations of inflammatory markers; (2) reviewed primary studies, including observational studies and/or intervention trials; and (3) were published in English in a peer-reviewed journal. Articles were excluded if the methodology was not conducted according to key characteristics of a systematic review.^32^^,^^34^ The authors were contacted for additional information if the required data were inadequately reported.

**Table 1.** PICOS Criteria for Inclusion of Review Studies

Parameter	Inclusion criterion	Exclusion criterion
Population	Human populations of all ages (no restrictions imposed on population characteristics: sex, age, health status)	(1) Animal populations; (2) in vitro
Phenomena of interest	Whole dietary patterns	(1) Isolated nutrients or food components, single foods or food groups; (2) intervention trials that combined other inextricable lifestyle modifications
Comparator	(1) Whole dietary patterns; (2) high/low adherence	
Context/setting	No restrictions imposed on geographical location, cultural, racial, or socioeconomic factors	Acute care
Outcome of interest	Measured concentrations of pro- and/or anti-inflammatory markers of chronic inflammation	Nonclinical outcomes
Study design	Systematic review and/or meta-analysis of intervention trials and/or observational studies	(1) Primary studies; (2) reviews with incomplete or inadequately defined methodology (ie, not conducted in accordance with defined systematic methodology); (3) includes primary intervention trials with a duration <4 weeks

### Study Selection

The identified articles were exported to Covidence (Covidence Systematic Review Software; Veritas Health Innovation, Melbourne, Australia. Available at: www.covidence.org) and duplicates were removed. Titles and abstracts were independently screened in duplicate according to the eligibility criteria by the authors (G.L.R., E.J.B., K.L.). When an abstract was unavailable or inadequate, the authors retrieved and examined the full text to determine eligibility. The remaining articles underwent full-text review and were further screened in duplicate against the eligibility criteria by authors (G.L.R., E.J.B., K.L.) independently. This process included an appraisal of each review synthesis and meta-analysis to determine whether the reported findings were derived from eligible primary studies. Meta-analyses that contained data extracted from any ineligible primary study were excluded from the current review. Similarly, narrative syntheses, based on ineligible primary studies, were also excluded. According to the PICOS criteria (**Table 1**), the primary studies were considered ineligible for the following reasons: (1) intervention trial with a duration of fewer than 4 weeks, set as the minimum to account for apparent changes in inflammatory marker concentrations^35^^,^^36^; (2) assessed the effects/association of single nutrients, food components, or food groups on inflammatory markers; and (3) intervention trials that included an inextricable combination of other lifestyle modifications such as exercise or counseling. All exclusions were documented for transparency and all discrepancies or disagreements were discussed and resolved by consensus.

### Data Extraction

Data extraction was performed by 1 reviewer (G.L.R.) and a 20% random sample of data^37^ were extracted and verified by 2 authors (E.J.B., K.L.) independently, with a minimum 80% consensus achieved.^38^ All extracted data were tabulated using a pre-piloted form based on the Cochrane Collaboration guidelines^39^ as follows: review author, published year, country, and funding; review type and objectives; database searches; characteristics of primary studies (design, duration, range in publication years, sample size, population age, sex, health status, and country); DPs and comparator diets assessed; primary and secondary outcomes; reported inflammatory markers; risk-of-bias assessment; publication bias; and review limitations. The results were derived from meta-analyses and narrative syntheses of included reviews and not from primary study-level data. For each review, all results for inflammation-related outcomes were extracted and reported. No imputations or assumptions were made for studies with missing or unclear data. If data were incomplete or ambiguous, such studies were either excluded from specific analyses or their available data were reported as presented in the original publication.

### Overlap of Primary Studies Assessment

Umbrella reviews present a unique challenge in addressing multiple reviews that overlap due to the inclusion of the same published primary studies.^40^^,^^41^ The potential for double-counting outcome data poses a risk for introducing bias by disproportionately weighting data from primary publications that are included in several reviews.^32^ The degree of overlap across the included reviews was documented and assessed by measurement of corrected covered area (CCA), a validated method to calculate the extent of overlap in an umbrella review.^40^ Where possible, the authors sought to avoid double-counting outcome data by using predefined criteria to prioritize the inclusion of the most recent or highest-quality review, or by ensuring that outcome data were extracted from overlapping reviews only once.^39^^,^^42^ The CCA was categorized and overlap interpreted as follows: slight (≤5), moderate (6–10), high (11–15), and very high (>15).^40^

### Methodological Quality Assessment

One author (G.L.R.) independently assessed the methodological quality of each included review using A Measurement Tool to Assess Systematic Reviews 2 (AMSTAR-2). Auditing was verified by the other authors, following the same protocol as that for data extraction.^38^ The 16-item AMSTAR-2 assists authors in identifying high-quality reviews, including those based on randomized controlled trials (RCTs) and nonrandomized studies of healthcare interventions, by assessing the extent to which review methodologies minimized critical and noncritical weaknesses.^38^ The AMSTAR-2 guidance document suggests critical domains that may adversely affect the validity of a review and must therefore be considered. These include: he protocol was registered prior to commencing review (item 2), included a comprehensive literature search strategy (item 4), listed and justified all excluded studies (item 7), assessed risk of bias in individual studies included in the review (item 9), used appropriate meta-analytical methods (item 11), accounted for risk of bias when interpreting review results (item 13) and, investigated/discussed publication bias (item 15).^38^ The overall quality was graded as follows: “high” if zero or only a single noncritical weakness, “moderate” if more than 1 noncritical weakness, “low” if 1 critical weakness with or without noncritical weaknesses, and “critically low” if more than 1 critical weakness with or without noncritical weaknesses.^38^ Discrepancies or disagreements were resolved by discussion to reach consensus.

### Data Synthesis

A narrative summary of outcome data was undertaken to describe and present the body of evidence on the association between DPs and inflammation.^32^ Where available, the effect estimates, 95% CIs, and measures of heterogeneity were extracted, grouped according to DPs, and visually presented.^32^ In instances where a review reported only the direction of effect/association (eg, systematic review without meta-analysis) or where there were inconsistencies in the data reported, vote counting was undertaken to summarize the data.^43^ The overall direction of the effect/association summary was calculated using the algorithm developed by Boon and Thomson^44^ as follows: if 70% or more (majority) of outcomes report a consistent direction of effect/association, then represent that direction (eg, “beneficial,” “harmful,” “no effect/association”); if less than 70% of outcomes report a consistent direction of effect/association or there are conflicting findings then represent a direction of “no effect/association.” In accordance with Cochrane methodological guidance, the purpose of vote counting is to evaluate the ratio of beneficial outcomes to unfavorable outcomes for a specific result.^43^ This alternate method of synthesis ensures a transparent link between data and conclusions in a systematic review without meta-analysis.^43^^,^^44^

### Certainty of Evidence

Where possible, Grading of Recommendations Assessment, Development, and Evaluation (GRADE) assessments, reported by the authors of the included reviews, are presented. In the absence of a reported GRADE assessment, the quality of evidence was assessed by 1 author (G.L.R.) and verified by a second author (E.J.B. or K.L.). According to the GRADE approach, the initial grading was based on the type of evidence, whereby intervention trials default to high certainty, whereas observational studies default to low certainty.^45^ Following this, each review was assessed using a GRADE algorithm developed for Cochrane Overviews of Reviews^46^^,^^47^ to objectively evaluate the strength of evidence and determine the overall GRADE level certainty of evidence (CoE). The algorithm derives the total number of downgrades against 4 key criteria: imprecision, risk of bias (primary studies), inconsistency (heterogeneity), and methodological quality of the review (eg, AMSTAR-2).^46^  **Table 2** presents the strength of evidence grades and definitions.^45^^,^^48^ Any disagreements were resolved by consensus.

**Table 2.** Strength of Evidence Grades and Definition

Grade	Definition
High	The body of evidence has few or no deficiencies. Further research is unlikely to change the conclusion.
Moderate	The body of evidence has some deficiencies. The findings are likely to be stable; however, some uncertainty remains.
Low	The body of evidence has major and/or numerous deficiencies. Further research is needed before concluding either that the findings are stable or close to the true effect/association.
Very low (insufficient)	The body of evidence has unacceptable deficiencies, and any estimate of effect/association is very uncertain.

Adapted from references ^45^ and ^48^.

## RESULTS

**Figure 1.**
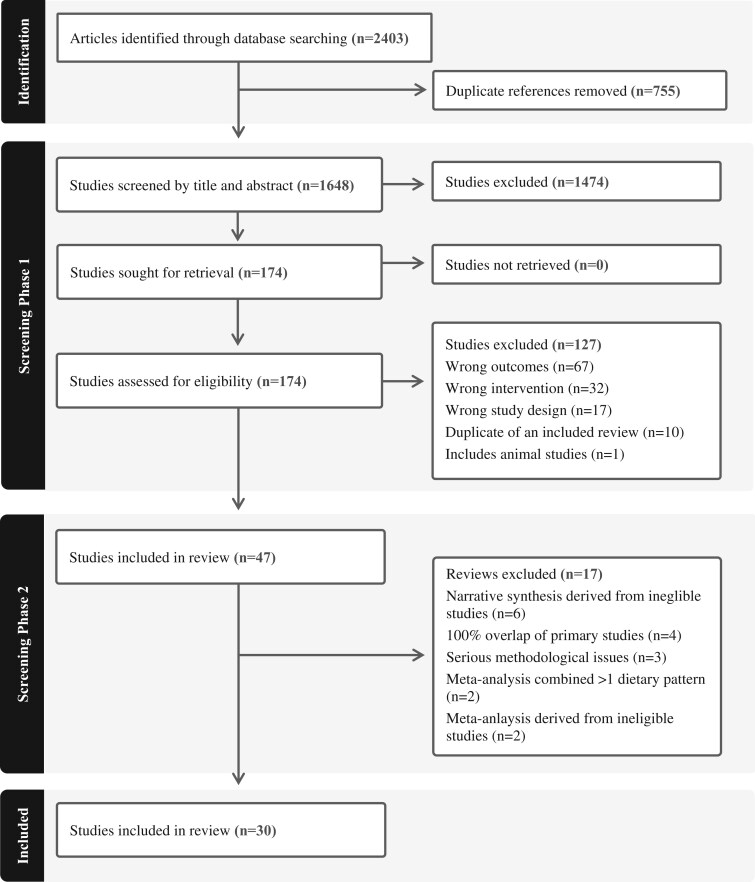
PRISMA (Preferred Reporting Items for Systematic Reviews and Meta-Analyses) Flow Diagram for the Selection of Reviews Describing Dietary Patterns Associated With Inflammation

**Figure 1** presents the PRISMA (Preferred Reporting Items for Systematic Reviews and Meta-Analyses) diagram of the comprehensive search of scientific databases and literature selection. A total of 2403 articles were retrieved, and after duplicates were removed, the remaining records (*n* = 1648) underwent title and abstract screening, with 1474 articles excluded due to failure to meet the eligibility criteria. Of the 174 studies that progressed to full-text review, 127 were excluded at screening phase 1 (**Figure 1**). Finally, the remaining 47 studies underwent screening phase 2, an in-depth appraisal of review syntheses and meta-analyses, and a further 17 reviews were excluded as follows: narrative synthesis derived from ineligible primary studies (*n* = 6),^29^^,^^49–53^ 100% overlap of eligible primary studies (*n* = 4),^54–57^ serious methodological issues (not conducted in accordance with Cochrane guidelines) (*n* = 3),^58–60^ meta-analysis combining more than 1 DPs (*n* = 2),^61^^,^^62^ and meta-analysis derived from ineligible primary studies (*n* = 2)^63^^,^^64^ (**Figure 1**). All exclusions related to this phase of study selection are presented in **Table S3**. Thirty reviews met the eligibility criteria and were included in the current umbrella review.

### Characteristics of Included Reviews

**Table 3** summarizes the 30 eligible review papers, including 9 systematic reviews and 21 systematic reviews with meta-analyses (SRMAs) published from 2011 to 2025, conducted in Australia,^36^^,^^65–68^ Germany,^69–72^ Canada,^73–75^ Iran,^76–80^ the United Kingdom,^81–83^ Austria,^15^^,^^23^ the United States,^84^^,^^85^ Cyprus,^86^ Greece,^87^ Italy,^88^ Mexico,^89^ Switzerland,^90^ and Taiwan.^91^ The proportion of primary studies meeting the inclusion criteria varied widely across the included reviews, ranging from 7% to 100%.^23^^,^^71^^,^^74^^,^^76^^,^^81^^,^^86^ The 225 unique primary studies comprised observational studies (*n* = 67) and experimental studies (*n* = 158). RCTs constituted the majority of studies (*n* = 146; 65%). Included primary studies were published between 1986 and 2024, with sample sizes ranging from 185 to 18 055 participants. Most reviews restricted their inclusion to adult populations (*n* = 25) and included both females and males (*n* = 29). Other reviews were more specific and included studies conducted only on children and adolescents,^83^ females aged 16–45 years,^85^ and elderly adults.^91^ Several individual reviews stipulated inclusion criteria relating to specific participant health status including the following: hyperlipidemia^74^; IBD in remission, diabetes (or risk factors) or CVD (or risk factors), coronary heart disease and/or a coronary event, overweight or obesity (OW/OB), coronary artery disease or OW/OB + 1 or more CVD risk factor, or T2D^89^; T1D or T2D^73^; T2D or gestational diabetes^82^; rheumatoid arthritis^86^; and pregnancy.^85^ Last, a single review included only studies conducted on healthy participants^71^ (**Table 3**).

**Table 3.** Characteristics of Included Reviews

Review type (country), funding source	Electronic databases, search time frame, participant inclusion criteria	Study characteristics: relevant studies, design, duration, publication range	Total sample size (range), sex, country	Range in health status of sample population	Dietary pattern assessed (variations in included comparator diets)	Primary outcome	**Reported inflammatory marker**	Limitations	AMSTAR-2 (rating of overall quality)
Aleksandrova, 2021^69^	Objective: To synthesize findings on associations between dietary patterns and markers of oxidative stress and inflammation								
Systematic review (Germany), no funding	Medline, PubMed, WoS Jan 2015–Oct 2020 ≥18 y	8 of 29 RCT: (*n* = 2); 2017–2020; 24–52 wk IT no comparison: (*n* = 2); 2019; 12–24 wk OB (CSS): (*n* = 4); 2016–2020	2155 (36–805) F, M AU, BR, CY, ES, GR, IR, IT, USA	OB; MetS; CVD NAFLD; healthy	1. MED (habitual; LFHC; adherence only) 2. VEG (omni) 3. HE (adherence only) 4. Paleo (adherence only)	Markers of oxidative stress and inflammation	CRP; IL-6; TNF-α; SAA	Heterogeneity/variability in markers and assessment measures; CSS study design limitations; low/moderate study quality in IT.	HIGH
Bujtor, 2021^83^	Objective: To synthesize the evidence on associations between dietary intake and low-grade inflammatory markers in children and adolescents								
Systematic review (UK), agency	CINAHL, Medline, PsycInfo, Embase, WoS Inception–Nov 2020 2–19 y	18 of 53 IT: (*n* = 5); 2010–2019; 6–24 wk OB: (*n* = 13); 2009–2016	6830 (22–2520) F, M AU, ES, GR, IR, IT, TR, USA	OW/OB; MetS; asthma; T1D; depression	1. MED (adherence only) 2. DASH (habitual) 3. HE (adherence only) 4. LG (high-glycemic) 5. High-protein (habitual) 6. Wstn (adherence only)	Inflammatory markers	CRP	Heterogeneity in study populations and lack of control for confounders. Potential for reporting bias.	MODERATE
Chiavaroli, 2018^74^	Objective: To evaluate available evidence of effect of the Portfolio diet on LDL-C and other risk factors for CVD prevention								
Systematic review and meta-analysis (Canada), agency	Medline, Embase, Cochrane (CENTRAL) Inception–Apr 2018 ≥18 y with HLD	5 of 5 RCT: (*n* = 7 trial comparisons); 2002–2011; 4–24 wk	435 (13–345) F, M CA	HLD	Portfolio (NCEP Step II)	LDL-C Secondary: blood lipids, adiposity, inflammation, BP, glycemic control	CRP	Serious imprecision (CRP outcomes); potential indirectness (trials conducted by a single investigator group) and did not include participants with T2D.	HIGH
Chiavaroli, 2021^73^	Objective: To summarize the effect of LG dietary patterns on glycemic control and other cardiometabolic risk factors in individuals with diabetes								
Systematic review and meta-analysis (Canada), institute	Medline, Embase, Cochrane (CENTRAL) Inception–May 2021 All ages + T1D/T2D (excl. pregnant)	6 of 27 RCT: (*n* = 6); 2008–2020; 3–5 wk	622 (20–210) F, M BR, CA, CN, IN, USA	Diabetes	LG (HG; low-fat; low-fiber; unspecified)	HbA1c Secondary: adiposity, BP, blood lipids, glycemic control, inflammation	CRP	Indirectness (lack of HG comparator diet); serious imprecision across outcomes, small number of trial comparisons for CRP; unable to perform publication bias analysis due to insufficient trial comparisons.	HIGH
Craddock, 2019^36^	Objective: To evaluate the modulating effects of vegetarian dietary patterns on inflammation or immune markers								
Systematic review and meta-analysis (Australia), no funding	Medline, PubMed, Cochrane (CENTRAL) Inception–Dec 2017 All ages. Adhered to VEG DP for ≤1 y	30 of 40 RCT: (*n* = 7); 1986–2015; 4–56 wk OB (CSS): (*n* = 23); 1990–2017	7633 (20–4109) F, M BR, CN, CL, CZ, FI, HK, IT, NG, NO, PL, SK, TH, TW, UK, USA	Dialysis; CVD; T2D; OW/OB; RA	1. VEG (omni) 2. VGN (omni)	CRP Secondary: other immune and inflammatory markers	CRP, IL-6, IL-10, TNF-α, fibrinogen	Limited studies, small sample sizes, and low quality of studies limited quantitative analysis. Lack of detail for diet type and quality. Lack of control for confounders. Limited generalizability (variations in study population and small sample sizes).	MODERATE
English, 2022^65^	Objective: To investigate the association between DPs and novel inflammatory markers: PAF and Lp-PLA2								
Systematic review (Australia), no funding	PubMed, Embase, CINAHL, ICTRP Cochrane (CENTRAL), CT.gov Inception–Feb 2021 ≥18 y	6 of 16 RCT: (*n* = 1); 2020; 52 wk IT: (*n* = 4); 2006–2018; 4–10 wk OB: (*n* = 1); 2011	914 (26–363) F, M CA, ES, GR, TW, USA	Healthy/T2D/CVD risk/MetS/HTN	1. MED (low-fat; usual)	Inflammatory markers	PAF, Lp-PLA_2_	Limited no. of studies; lack of consensus for assessment and cutoff points for markers; risk of bias and variability in study populations.	MODERATE
					2. VEG (omni)				
					3. VGN (adherence only)				
Grammatikopoulou, 2020^87^	Objective: To assess and update the evidence on the effectiveness of the low-FODMAP diet in patients with IBD and FGD								
Systematic review (Greece), no funding	PubMed, Scopus, Cochrane (CENTRAL), CT.gov Inception–Apr 2020 All ages with IBD in remission	3 of 4 RCT: (*n* = 3); 2014–2019; 4–6 wk	185 (52–78) F, M DK, IT, UK	IBD: remission	Low-FODMAP (habitual; sham exclusion)	Symptom severity, markers of immunity and inflammation	CRP	Bias inherent to nutrition interventions in IBD (placebo response phenomenon); low adherence to long-term elimination diet; lacked composite endpoints.	HIGH
Haghighatdoost, 2017^78^	Objective: To investigate the effects of a vegetarian DP (compared with omnivore DP) on inflammatory markers								
Systematic review and meta-analysis (Iran), no funding	Science Direct, Proquest, Medline, Google Scholar Inception–June 2016 ≥18 y	17 of 18 OB (CSS): (*n* = 17); 1999–2016; 6 mo to >5 y	2398 (36–363) F, M CL, CN, IC, DE, IT, SK, TW, USA	Not reported	VEG (omni)	Inflammatory markers	CRP, IL-6	Limited studies on IL-6; based on CSS data only; no intake assessment for comparator DP; limited generalizability due to DP variations across studies; lack of food preparation methods assessment.	CRITICALLY LOW
Ji, 2025^80^	Objective: To evaluate the effects of the ketogenic diet on inflammatory biomarkers								
Systematic review and meta-analysis (Iran), no funding	PubMed/Medline, Scopus, Web of Science, EMBASE Inception–Aug 2023 ≥18 y	21 of 41 RCT: (*n* = 21); 2006–2023; 6–54 wk	1081 (15–263) F, M AU, CA, CZ, DE, NZ, UK, USA	OW, OB	Ketogenic (habitual; high-carb; low-fat)	Inflammatory markers	CRP, TNF-α, IL-6, IL-8	Small number of studies; short study duration; sources of heterogeneity not explained; limited generalizability (OW/OB).	LOW
Koelman, 2022^70^	Objective: To summarize the recent evidence on the effects of dietary patterns on immune-related and inflammatory markers								
Systematic review and meta-analysis (Germany), no funding	PubMed, Medline, WoS Jan 2015–Oct 2020 ≥18 y	22 of 23 RCT: (*n* = 22); 2015–2020; 4 wk–5 y	2746 (23–897) F, M AU, BR, DK, ES, GR, IR, IT, PL, SE, UK, USA	CVD or risk factors; T2D; OB	1. MED (low-fat; habitual) 2. VEG/VGN (omni; AHA) 3. DASH (habitual)	Inflammatory markers	CRP; IL-6; TNF-α; IL-8; E-selectin; IFN-γ; IL-1β	Limited to CRP and may not capture full spectrum of inflammation in NCD; limited generalizability (>50 y); high heterogeneity and a lack of investigation for potential sources.	MODERATE
Massara, 2022^75^	Objective: To inform the updated clinical practice guidelines for nutrition therapy, on Nordic DPs and cardiometabolic outcomes								
Systematic review and meta-analysis (Canada), agency	Embase, Medline, Cochrane (CENTRAL) Library Inception–Mar 2021 Adults with diabetes/risk factors or CVD/risk factors	5 of 21 RCT: (*n* = 5); 2008–2020; 6–26 wk	606 (86–166) F, M DK, FI, IS, SE	T2D or CVD risk factors: OB/OW; MetS; DLD	Nordic (habitual; healthy eating)	LDL-C Secondary: lipid targets, BP markers of glycemic control, inflammation	CRP	Serious inconsistencies and imprecision; substantial unexplained heterogeneity in outcomes; indirectness (variation in DP definition); indirectness (lack of data on T2D status).	HIGH
Mayr, 2018^66^	Objective: To compare the efficacy and safety of LC diets in relation to LF diets in individuals with T2D								
Systematic review and meta-analysis (Australia), no funding	PubMed, WoS, Medline, Embase, CT.gov, Cochrane (CENTRAL) Jan 1981–Jul 2021 ≥18 y, CHD or event	8 of 11 RCT: (*n* = 3); 2008–2015; 6–52 wk IT: (*n* = 1); 2011; 12 wk OB (CSS, PCS): (*n* = 4); 2005–2016	18 055 (24–15 482) F, M BR, ES, EU, IR, USA	CHD	MED (low-fat; TLC; AHA; Wstn; adherence only)	Inflammatory markers	CRP; IL-6; MCP-1; TNF-α; E-selectin	Limited sample sizes, inconsistent approaches, lack of diverse outcome measures limitations in data analysis and study pooling; lack of studies assessing inflammatory markers as primary outcome.	MODERATE
Menzel, 2020^71^	Objective: To evaluate the effects of vegetarian or vegan DPs on inflammatory markers, in both healthy and diseased populations								
Systematic review and meta-analysis (Germany), no funding	Embase, PubMed Inception–Apr 2020 ≥18 y, apparently healthy	21 of 21 OB (CSS): (*n* = 21); 1999–2020; 1–25 y	2058 (28–4109) F, M BR, CL, CN, DE, IN, IT, KR, SK, TW, UK	Apparently healthy	1. VEG (omni) 2. VGN (veg; omni)	Markers of inflammation	CRP, E-selectin, ApN	Limited to CSS; high heterogeneity (vegetarian DPs); small sample sizes; methodological variability; variability in assay quality measures and selection of inflammatory marker.	MODERATE
Moore, 2022^81^	Objective: To evaluate the effects of a Mediterranean DP on BMI and inflammatory markers in adults with OW/OB and at risk of developing severe COVID-19 outcomes								
Systematic review (UK), no funding	PubMed, Cochrane (CENTRAL), Medline Jan 2010–Aug 2021 ≥18 y, OW/OB	6 of 6 RCT: (*n* = 3); 2013–2019; 12–52 wk IT: (*n* = 3); 2011–2019; 8–12 wk	419 (36–129) F, M BR, ES, IT, USA	OB/OW	MED (TLC; MyPyramid; veg; keto; adherence only)	BMI, inflammatory markers	CRP; IL-6; TNF-α; IL-10	Limited generalizability (postnatal/breastfeeding participants); lack of randomization; short duration of studies (≥8 wk); bias and inconsistencies in data.	MODERATE
Mukherjee, 2023^68^	Objective: To assess the effects of anti-inflammatory diets, and their constituents thereof, on inflammatory markers								
Systematic review (Australia), no funding	Medline, PubMed, EMCare, Cochrane (CENTRAL), CINAHL Inception–Aug 2020 ≥18 y	14 of 20 RCT: (*n* = 14); 2005–2019; 3 mo–5 y	2023 (33–772) F, M AU, DZ, ES, PL, UK, USA	T2D or HTN or DLD; CAD; OA; CRF; Cx	MED (low-fat; habitual)	Inflammatory markers	CRP; IL-6; TNF-α; IL-10	Variability in inflammatory markers measured; unable to conduct meta-analyses (high heterogeneity in study population, interventions); lack of data on outcomes; overlap in data.	HIGH
Neale, 2016^67^	Objective: To determine the effect of healthy DPs on markers of adiposity, IR, and inflammation in adults								
Systematic review and meta-analysis (Australia), no funding	Scopus, PubMed, WoS, Cochrane (CENTRAL) Inception–Apr 2015 ≥18 y	15 of 17 RCT (*n* = 15); 2003–2014, 4–104 wk	1345 (11–204) F, M AU, DK, DZ, ES, FI, GR, IS, IL, IT, NZ, SE, USA	Healthy or OW/OB	1. MED (habitual; LFHC; low-fat; low-carb; healthy eating) 2. Nordic (habitual)	Inflammatory markers	CRP; TNF-α; resistin; ApN	Small no. of studies; limited generalizability of findings; variability in DP definitions.	MODERATE
Nordmann, 2011^90^	Objective: To synthesize the evidence from RCTs with a minimum duration of 6 months comparing the effects of a Mediterranean DP with a low-fat DP on CVD risk factors in individuals with OW/OB								
Systematic review and meta-analysis (Switzerland), no funding	Medline, Embase, PubMed, WoS, Cochrane (CENTRAL) Inception–Jan 2011 ≥18 y, CAD or OW/OB + ≥1 CVD	5 of 7 RCT: (*n* = 5); 2003–2008; 2–6 y	2435 (101–1821) F, M Country unspecified	OW/OB; CVD	MED (low-fat)	Markers of CVD risk	CRP	Small no. of trials; lack of power to detect clinical differences in outcomes; high heterogeneity; limited generalizability; did not investigate effect of individual DP components on CVD risk.	LOW
Ojo, 2019^82^	Objective: To evaluate the effects of an LG DP on cardiometabolic and inflammatory parameters in patients with GDM and T2D								
Systematic review and meta-analysis (UK), no funding	Embase, PubMed, PsycINFO Inception–Feb 2019 ≥18 y, T2D or GDM	5 of 9 RCT: (*n* = 5); 2008–2017; 4–52 wk	420 (20–162) F, M BR, CA, CN, GR	T2D; GDM	LG (HG)	Inflammatory markers	CRP; IL-6; ApN	Variability in DP definition	LOW
Philippou, 2021^86^	Objective: To update and inform the current literature and to gain better insights into the role of diet on RA outcomes								
Systematic review (Cyprus), no funding	Medline, Embase Inception–Oct 2018 ≥18 y, rheumatoid arthritis (RA)	5 of 70 RCT: (*n* = 5); 2002–2008; 4–52 wk	311 (22–100) F, M Country NR	RA	1. MED (adherence only) 2. VGN (omni; low-fat)	RA outcomes	CRP	Small sample sizes; high attrition; lacked intention-to-treat analysis; potential bias (placebo effect); unable to conduct meta-analyses (high heterogeneity).	LOW
Pickworth, 2019^84^	Objective: To assess the association between dietary patterns and hs-CRP among enrolled individuals								
Systematic review (USA), no funding	Medline, Google Scholar Jan 2000–Oct 2017 No restrictions on age or health status	29 of 56 RCT: (*n* = 29); 2004–2016; 4–104 wk	3539 (29–772 F, M AU, CA, DE, DK, ES, EU, GR, IR, IL, IT, SE, USA	CVD risk; MetS; OB/OW; T2D	1. MED (healthy eating; low- fat) 2. LG (high-glycemic; habitual) 3. LC (LFHC; habitual) 4. HP (habitual)	CRP	CRP	Variability in study populations and study characteristics; high risk of bias.	LOW
Pourrajab, 2025^79^	Objective: To evaluate the effects of Mediterranean diet in comparison to the low-fat diet on inflammation and endothelial indexes in adults								
Systematic review and meta-analysis (Iran), no funding	PubMed/Medline, Scopus, Web of Science, Cochrane (CENTRAL), Google Scholar Inception–Aug 2024 ≥18 y	15 of 16 RCT: (*n* = 15); 2001–2023; 4–240 wk	3455 (32–805) F, M AU, ES	CVD risk; T2D; CHD, NAFLD, HTN, HCL	High-fat MED + EVOO (low fat)	Inflammatory markers, endothelial indexes	CRP; IL-6; TNF-α; ICAM-1; VCAM-1; selectin	Small sample sizes for some inflammatory markers; publication bias detected; sources of heterogeneity not explained; limited generalizability to Spain, populations at risk of CVD.	HIGH
Rondanelli, 2024^88^	Objective: To assess the effects of the ketogenic diet on inflammatory biomarkers in individuals with OW/OB								
Systematic review and meta-analysis (Italy), no funding	PubMed, Scopus, Web of Science, Google Scholar Jan 2013–Jul 2024 ≥18 y	5 of 7 RCT: (*n* = 5); 2016–2024; 2–6 mo	167 (16–57) F, M NR	OB, OW	Ketogenic: ∼15% carb; ∼60% fat; ∼25% protein (WFD; MED; VLC)	Markers of inflammation	CRP, IL-6	Short study duration; unexplained heterogeneity, small sample sizes, lack of standardized protocols, potential publication bias, inadequate reporting of adverse events.	LOW
Sakhaei, 2019^76^	Objective: To summarize the evidence on the effect of the Nordic DP on inflammatory markers								
Systematic review and meta-analysis (Iran), no funding	PubMed, Scopus, WoS, Google Scholar Inception–Oct 2017 ≥18 y	7 of 7 RCT: (*n* = 7); 2011–2017; 6–26 wk	613 (70–199) F, M DK, FI, IS, SE	OB; MetS; HLC; postmenopausal	Nordic (habitual; Paleo)	Markers of inflammation	CRP	Short study duration; unexplained heterogeneity.	HIGH
Sánchez-Rosales, 2022^89^	Objective: To explore the inflammatory effect of healthy DPs on inflammatory markers in adults with T2D								
Systematic review and meta-analysis (Mexico), no funding	Medline, Scopus, Cochrane (CENTRAL) Inception–Jan 2022 ≥18 y with T2D	4 of 10 RCT: (*n* = 4); 2011–2016; 24 wk–8 y	479 (27–215) F, M AU, ES, IL, IT	T2D	MED (low-fat; habitual)	Markers of inflammation Secondary: glycemic control	CRP, ApN	Lack of standardization of inflammatory markers; limited no. of studies; high heterogeneity in DPs assessed; quantitative variables and small sample sizes.	MODERATE
Schwingshackl, 2013^15^	Objective: To investigate long-term (6 months) effects of glycemic-related diets in the management of obesity and their potential usability in prevention of obesity-associated disorders								
Systematic review and meta-analysis (Austria), no funding	Medline, Embase, Cochrane (CENTRAL) 1980–-Feb 2013 ≥18 y with OW/OB	7 of 14 RCT: (*n* = 7); 2008–2011; 24–52 wk	1648 (108–619) F, M Country unspecified	OB/OW	LG (HG; HPLF; low-fat; high-fiber)	Obesity-related markers	CRP	High heterogeneity (study and population characteristics); variability in GI/GL values; insufficient data on study quality; potential publication bias.	CRITICALLY LOW
Schwingshackl, 2014^23^	Objective: To summarize the available evidence regarding the effect of a Mediterranean DP on outcomes of endothelial function and inflammation								
Systematic review and meta-analysis (Austria), no funding	Medline, Embase, Cochrane (CENTRAL) Inception–Feb 2014 ≥19 y	17 of 17 RCT: (*n* = 17); 2003–2013; 3–48 mo	2300 (23–772) F, M Country unspecified	OB; MetS; CVD risk; T2D	MED (habitual; low-fiber; low-fat; healthy eating)	Markers of inflammation and endothelial function	CRP; IL-6; E-selectin; ICAM-1; ApN	Lack of power (inflammatory marker outcomes); high heterogeneity (study population and duration), variability in DP definitions and intake assessment methods.	LOW
Soltani, 2018^77^	Objective: To summarize the evidence from RCTs examining the effect of DASH DP on inflammatory in adults								
Systematic review and meta-analysis (Iran), no funding	Scopus, Embase, PubMed, Google Scholar Inception–Dec 2016 ≥18 y	4 of 7 RCT: (*n* = 4); 2011–2015; 8–24 wk	451 (31–241) F, M Country unspecified	T2D; PCOS; NAFLD; HLD	DASH (habitual; portfolio)	Markers of inflammation	CRP	Lack of control for potential confounders (weight change) variability in DP adherence; potential bias (crossover design); limited generalizability and small no. of studies.	HIGH
Steckhan, 2016^72^	Objective: To assess the effects of dietary interventions on markers of inflammation in individuals with MetS								
Systematic review and meta-analysis (Germany), foundation	Medline, Scopus, Cochrane (CENTRAL) Inception–Sep 2014 ≥18 y + MetS	3 of 13 RCT: (*n* = 3); 2004–2009; 12–52 wk	289 (39–132) F, M AU, AE, USA	MetS	1. LC (LFHC; habitual) 2. LF (low-carb)	Markers of inflammation	CRP	Limited generalizability. Unable to conduct MA (small no. of studies, high heterogeneity); variability in comparator diets and baseline characteristics; intra- and interindividual biological variability inherent to inflammatory markers; lack of allocation concealment and assessor blinding; median dropout rate.	LOW
Wu, 2021^91^	Objective: To explore the association between the Mediterranean DP and inflammation in older adults								
Systematic review and meta-analysis (Taiwan), no funding	PubMed, Embase, Scopus, WoS, Cochrane (CENTRAL), CINAHL, ProQuest Inception–Jun202 ≥65 y	8 of 13 RCT: (*n* = 2); 2006–2009; 3 mo OB (CSS): (*n* = 6); 2010–2019	9512 (194–2646) F, M CN, DE, ES, EU, IT, NO, UK, USA	CAD + elderly	MED (low-fat; habitual; adherence only)	Markers of inflammation	CRP; CRP; IL-6; TNF-α	Small no. of studies; variability in study quality, populations, and methods; high heterogeneity; lack of control for confounders; limited to a few inflammatory markers; limited generalizability (community-dwelling older adults).	MODERATE
Yeh, 2021^85^	Objective: To synthesize the current evidence on the relationship between DPs and inflammatory markers during pregnancy								
Systematic review (USA), no funding	Embase, PubMed, Scopus, WoS Inception–May 2020 Female + pregnant, 16-45 y	5 of 17 RCT: (*n* = 2); 2016–2019; 4–20 wk OB (CSS, PCS): (*n* = 3); 2010–2019	1495 (100–621) F AU, BR, LB, USA	Pregnant; GDM risk factors; macrosomia risk; healthy	1. MED (adherence only) 2. LG (HG; high-fiber; habitual)	Markers of inflammation	CRP, IL-6, TNF-α	Measurement bias (dietary assessment); variability in study design, DPs and definitions; lack of consensus on gestational inflammatory markers; high heterogeneity (methodological and statistical).	CRITICALLY LOW

Country abbreviations: AE, United Arab Emirates; AU, Australia; BR, Brazil; CA, Canada; CL, Chile; CN, China; CY, Cyprus; CZ, Czech Republic; DE, Germany; DK, Denmark; DZ, Algeria; ES, Spain; EU, Europe; FI, Finland; GR, Greece; HK, Hong Kong; IC, Ivory Coast; IL, Israel; IN, India; IR, Iran; IS, Iceland; IT, Italy; KR, Korea; LB, Lebanon; NG, Nigeria; NO, Norway; NZ, New Zealand; PL, Poland; SE, Sweden; SK, Slovakia; TH, Thailand; TR, Turkey; TW, Taiwan.

Dietary pattern abbreviations: AHA, American Heart Association diet; DASH, Dietary Approaches to Stop Hypertension diet; FODMAP, fermentable oligosaccharides, disaccharides, monosaccharides, and polyols; HE, healthy eating; HG, high-glycemic; HPLF, high-protein low-fat; keto, ketogenic; LC, low-carbohydrate; LF, low-fat; LFHC, low-fat high carbohydrate; LG, low-glycemic; MED, Mediterranean; NCEP, National Cholesterol Education Program Step II diet; omni, omnivorous; Paleo, Paleolithic diet; TLC, Therapeutic Lifestyle Change diet; VEG, vegetarian; VGN, vegan; Wstn, Western.

Other abbreviations: AMSTAR-2, A Measurement Tool to Assess Systematic Reviews 2; ApN, adiponectin; BMI, body mass index; BP, blood pressure; CAD, coronary artery disease; CENTRAL, Cochrane Central Register of Controlled Trials; CHD, coronary heart disease; CRF, chronic renal failure; CRP, C-reactive protein; CS, cohort study; CSS, cross-sectional study (studies); CT.gov, ClinicalTrials.gov; CVD, cardiovascular disease; Cx, cancer; DP, dietary pattern; DLD, dyslipidemia; EVOO, extra-virgin olive oil; F, female; FGD, functional gastrointestinal disorders; GDM, gestational diabetes; HbA1c, glycated hemoglobin; HCL, hypercholesterolemia; HF, heart failure; HLD, hyperlipidemia; hs-CRP, high-sensitivity C-reactive protein; HTN, hypertension; IBD, inflammatory bowel disease; ICAM, intracellular adhesion molecule 1; ICTRP, International Clinical Trials Registry Platform; IFG, impaired fasting glucose; IFN-γ, interferon gamma; IL, interleukin; IT, intervention trial(s); LDL-C, low-density-lipoprotein cholesterol; Lp-PLA_2_, lipoprotein-associated phospholipase A2; LS, longitudinal study; M, male; MA, meta-analysis; MetS, metabolic syndrome; MS, multiple sclerosis; NAFLD, nonalcoholic fatty liver disease; OA, osteoarthritis; OB, obese; OW, overweight; PAF, platelet-activating factor; PCS, prospective cohort study; RA, rheumatoid arthritis; SAA, serum amyloid A; T1D, type 1 diabetes; T2D, type 2 diabetes; TNF-α, tumor necrosis factor alpha; VCAM-1, vascular cell adhesion molecule 1; VLC, very low calorie; WFD, whole-food diet; WoS, Web of Science.

The majority of the examined reviews did not specify the methods used to assess dietary intake and adherence. Of the reviews that did provide this information, food-frequency questionnaires (FFQs), 24-hour recall (24HR). and 3-day food records were the most commonly reported methods^66^^,^^68^^,^^69^^,^^73^^,^^79^^,^^81^^,^^85–87^^,^^89^^,^^91^ (**Table S4**). Furthermore, the methods used to construct dietary patterns were not described or accounted for in any meta-analysis included in this review.

A total of 15 DPs were assessed including the following: Mediterranean (*n* = 16),^23^^,^^65–70^^,^^79^^,^^81^^,^^83^^,^^84–86^^,^^89–91^ vegetarian (*n* = 6),^36^^,^^65^^,^^69–71^^,^^78^ low-glycemic (LG; *n* = 6),^15^^,^^73^^,^^82–85^ vegan (*n* = 4),^36^^,^^65^^,^^71^^,^^86^ DASH (*n* = 3),^70^^,^^77^^,^^83^ Nordic (*n* = 3),^67^^,^^75^^,^^76^ Healthy Eating, based on Dietary Guidelines for Americans (HE; *n* = 2),^69^^,^^83^ low-carbohydrate (LC; *n* = 2),^72^^,^^84^ high-protein (HP; *n* = 2),^83^^,^^84^ ketogenic (*n* = 2),^80^^,^^88^ low-fat,^72^ low-FODMAP (fermentable oligosaccharides, disaccharides, monosaccharides, and polyols),^87^ Portfolio,^74^ Paleolithic,^69^ and Western DPs.^83^ The majority of the included SRMAs did not provide comprehensive definitions or descriptions of the DPs they assessed. Based on their dietary composition and underlying principles, the Mediterranean, vegetarian, vegan, DASH, Nordic, HE, and Portfolio DPs predominantly emphasize plant foods. These plant-centric DPs are characterized by a high intake of fruits, vegetables, whole grains, legumes, nuts, and seeds.^92^ They are typically rich in fiber, antioxidants, and unsaturated fats.^93^^,^^94^ Most, except for vegan and some vegetarian variations, include fish, poultry, and low-fat dairy to varying degrees.^92^^,^^95^ For instance, the Mediterranean and Nordic diets emphasize regular fish consumption, whereas the DASH diet includes lean meats and low-fat dairy.^92^^,^^96^

**Figure 2.**
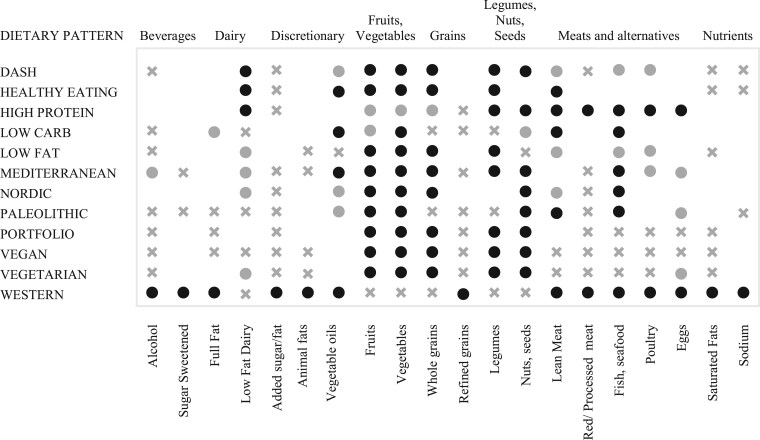
Comparison of Dietary Composition and Categorization of Foods and Food Groups Within Dietary Patterns. Black circle indicates foods that are emphasized or encouraged as part of a healthy diet. Grey circle denotes foods that are included in moderate amounts. Grey cross indicates foods that are restricted or limited. Abbreviation: DASH, Dietary Approaches to Stop Hypertension

There are several unique characteristics that differentiate the reviewed DPs: extra-virgin olive oil, rich in monounsaturated fats and antioxidants, is a cornerstone of the Mediterranean diet, serving as the primary source of dietary fat^92^^,^^97^; the low-FODMAP and LG diets emphasize food selection based on specific carbohydrate characteristics within broader food groups, focusing on fermentable carbohydrate content and glycemic index, respectively^15^^,^^73^^,^^98^; the ketogenic diet is very low in carbohydrates (*<*10% of total energy) and high in fats (*>*45% of total energy)^80^^,^^88^; the Portfolio diet combines cholesterol-lowering foods, such as plant proteins, viscous fiber, nuts, and phytosterols^99^^,^^100^; and the vegan diet excludes all animal products.^101^ A detailed comparison of the dietary composition of these DPs is shown in **Figure 2**.

The included reviews demonstrated considerable variation in comparator diets. Several meta-analyses aggregated data from primary studies comparing the Mediterranean DP with low-fat, habitual, or healthy diets.^23^^,^^67^^,^^70^^,^^91^ However, few reviews conducted meta-analyses that stratified results based on the comparator diet. Specific meta-analyses evaluated the relationship between inflammation and the Mediterranean DP in comparison to a low-fat diet,^66^^,^^89^^,^^90^ an LG diet compared with a high-glycemic diet,^82^ and a Portfolio DP in comparison to the National Cholesterol Education Program (NCEP) Step II diet.^74^

Inflammatory markers were the primary outcome for all reviews except for 3.^73–75^ C-reactive protein was assessed in all but 1 review,^65^ whereas other proinflammatory markers, including IL-6 and TNF-α, were assessed in 17 reviews (**Table 3**). The anti-inflammatory marker adiponectin was assessed in 5 reviews.^23^^,^^67^^,^^71^^,^^82^^,^^89^ A single review assessed platelet-activating factor and lipoprotein-associated phospholipase A2 and was the only included review to investigate studies that used novel inflammatory marker outcomes.^65^

### Overlap

**Figure 3.**
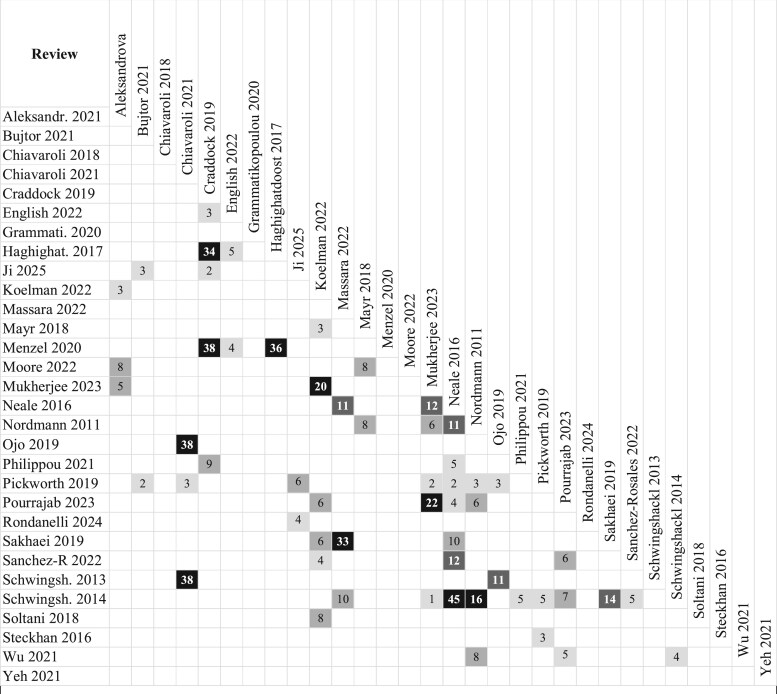
Citation Matrix Presenting the Cross-sectional Overlap (Percentage) Between Individual Reviews. Shading indicates the extent of overlap (calculated by CCA): black, very high overlap (>15%); dark grey, high overlap (11%–15%); lighter grey, moderate overlap (6%–10%); lightest grey, slight overlap (0%–5%). Abbreviations: Aleksandr, Aleksandrova; CCA, corrected covered area; Haghighat, Haghighatdoost; Sanchez-R, Sánchez-Rosales; Schwingsh, Schwingshackl

The overall extent of overlap (assessed using CCA) was slight (1.4%). Additionally, overlap was calculated for each DP as follows: high for vegan and Nordic; moderate for vegetarian; slight for DASH, Mediterranean, ketogenic, and LG; and nil for all other DPs. **Figure 3** illustrates the cross-sectional overlap among individual reviews, representing the percentage of shared primary studies. This overlap indicates commonality in the referenced literature across different reviews.

### Methodological Quality

Methodological quality, assessed by the AMSTAR-2 critical appraisal, was evaluated as high in 30% (*n* = 9), moderate for 33% (*n* = 10), low in 27% (*n* = 8), and critically low in 10% (*n* = 3) of the included reviews (**Figure S1**). All included reviews met 8 of the 16 quality appraisal criteria and, where applicable, satisfied 5 of the 7 critical domains (items 4, 7, 9, 11, and 15) (**Figure S2**). The most overlooked domains were reporting of the funding source of the included primary studies (item 10), performing study selection in duplicate (item 5), and performing data extraction in duplicate (item 6) (**Figure S2**).

### Summary of Synthesis and Strength of Evidence for DPs on Inflammation

Among the 21 SRMAs, 60 individual pooled analyses (unique meta-analyses) met the eligibility criteria for inclusion. Most reviews conducted random-effects meta-analyses to calculate the overall weighted mean differences (WMDs), with 95% CIs for outcome values. A single review applied the fixed-effects model,^82^ while 5 reviews calculated the overall standardized mean difference (95% CI).^72^^,^^78^^,^^79^^,^^89^^,^^91^

**Figure 4.**
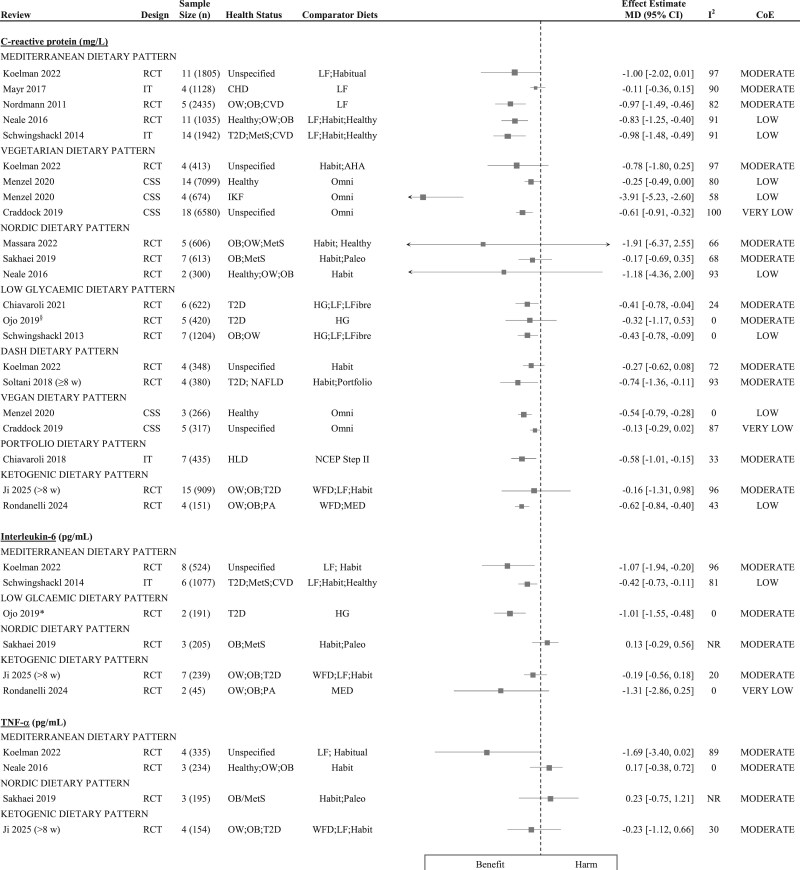
Forest Plots Summarizing the Meta-analyses That Investigated the Relationship Between Dietary Patterns and Inflammatory Markers, Reported as MD with 95% CIs. Each effect estimate represents the pooled MD for a specific proinflammatory marker, comparing a dietary pattern with a comparator diet. Negative MD values indicate a reduction in the proinflammatory marker, suggesting a potential beneficial effect/association, while positive values indicate an increase in the proinflammatory marker, suggesting a potential harmful effect/association. *The listed comparator diets encompass all diets evaluated against the intervention dietary pattern, across all primary studies included in the meta-analysis. ^§^Indicates meta-analyses that applied a fixed-effects model. Abbreviations: AHA, American Heart Association; CHD, coronary heart disease; CoE, certainty of evidence; CSS, cross-sectional study; CVD, cardiovascular disease; GDM, gestational diabetes mellitus; Habit, habitual; HG, high-glycemic; HLD, hyperlipidemia; IKF, impaired kidney function; IT, intervention trial; LF, low-fat; LFiber, low-fiber; MD, mean difference; MED, Mediterranean dietary pattern; MetS, metabolic syndrome; NAFLD, nonalcoholic fatty liver disease; NCEP, National Cholesterol Education Program; OB, obesity; Omni; omnivorous; OW, overweight; Paleo, Paleolithic; PA, psoriatic arthritis; RCT, randomized controlled trial; T2D, type 2 diabetes; TNF-α, tumor necrosis factor alpha; WFD, whole food diet

**Figure 5.**
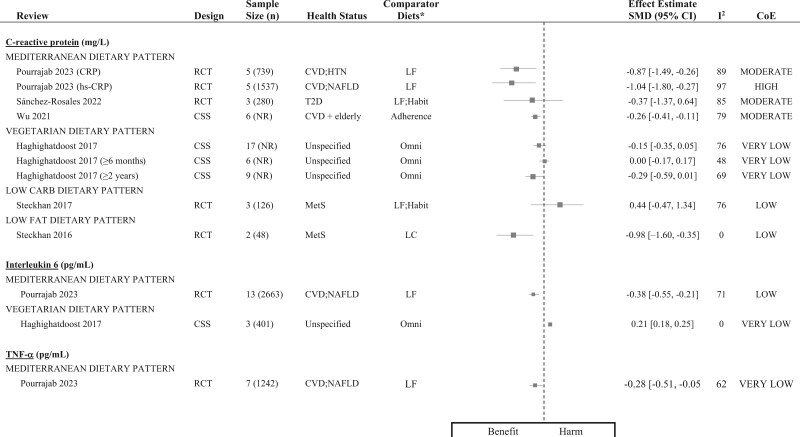
Forest Plots Summarizing the Meta-analyses That Investigated the Relationship Between Dietary Patterns and Inflammatory Markers, Reported as SMD with 95% CIs. Each effect estimate represents the pooled SMD for a specific proinflammatory marker, comparing a dietary pattern with a comparator diet. Negative SMD values indicate a reduction in the proinflammatory marker, suggesting a potential beneficial effect/association, while positive values indicate an increase in the proinflammatory marker, suggesting a potential harmful effect/association. *The listed comparator diets encompass all diets evaluated against the intervention dietary pattern, across all primary studies included in the meta-analysis. Abbreviations: CARB, carbohydrate; CoE, certainty of evidence; CRP, C-reactive protein; CSS, cross-sectional study; CVD, cardiovascular disease; Habit, habitual; hs-CRP, high-sensitivity C-reactive protein; HTN, hypertension; LC, low-carbohydrate; LF, low-fat; MetS, metabolic syndrome; NAFLD, nonalcoholic fatty liver disease; NR, not reported; Omni; omnivorous; RCT, randomized controlled trial; SMD, standardized mean difference; T2D, type 2 diabetes; TNF-α, tumor necrosis factor alpha

**Figure 6.**
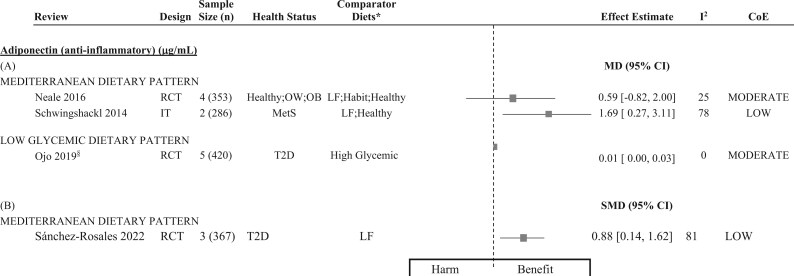
Forest Plots Summarizing the Meta-analyses That Investigated the Relationship Between Dietary Patterns and Inflammatory Markers, Reported as (A) MD (95% CI) and (B) SMD (95% CI). Each effect estimate represents the pooled MD or SMD for adiponectin, comparing a dietary pattern with a comparator diet. Negative values indicate a reduction in the anti-inflammatory marker, suggesting a potential harmful effect/association, while positive values indicate an increase in the anti-inflammatory marker, suggesting a potential beneficial effect/association. *The listed comparator diets encompass all diets evaluated against the intervention dietary pattern, across all primary studies included in the meta-analysis. ^§^Indicates meta-analysis that applied a fixed-effects model. Abbreviations: CoE, certainty of evidence; Habit, habitual; IT, intervention trial; LF, low-fat; MD, mean difference; MetS, metabolic syndrome; OB, obesity; OW, overweight; RCT, randomized controlled trial; SMD, standardized mean difference; T2D, type 2 diabetes

**Figure 7.**
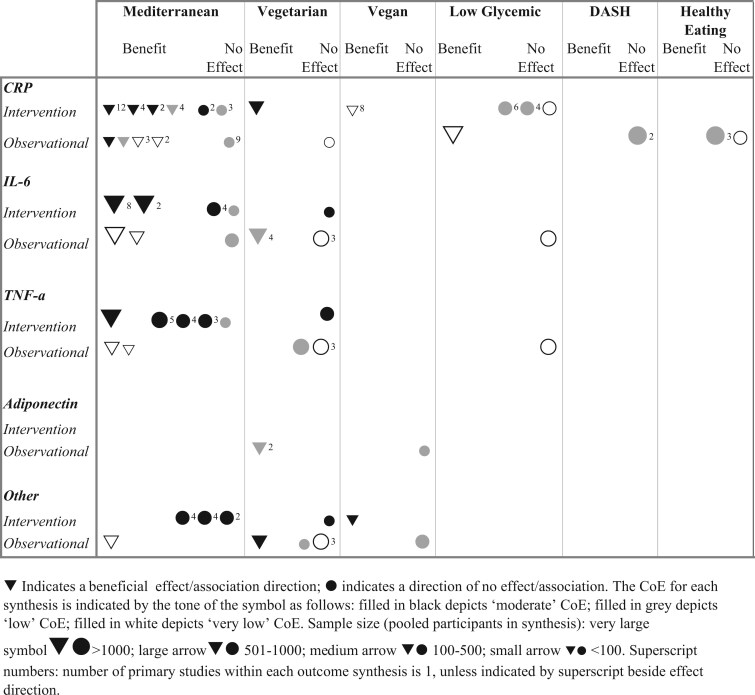
Summary of the Direction of Effect (for Intervention Trials) and the Direction of Association (for Observational Studies) on Inflammatory Marker Outcomes. The direction of effect/association is determined as follows:“beneficial” or “no effect/association” is indicated when ≥70% of outcomes reported a consistent direction; “no effect/association” is indicated when <70% of outcomes were consistent or findings conflict. Abbreviations: CoE, certainty of evidence; CRP, C-reactive protein; DASH, Dietary Approaches to Stop Hypertension; IL-6, interleukin 6; TNF-α, tumor necrosis factor alpha

**Figures 4–6** present forest plots summarizing the effect estimates of the relationship between DPs and key inflammatory markers. **Table S5** shows a complete report of all individual meta-analyses included in this review, detailing their characteristics, pooled effect estimates, and CoE for the relationship between dietary patterns and assessed inflammatory biomarkers. Additionally, 61 narrative syntheses were included in this review and the summarized direction of effect/association for key DPs is presented in **Figure 7**.

The overall CoE, as assessed using GRADE, varied across narrative syntheses and meta-analyses. For meta-analyses, the confidence ratings were moderate (52%), low (30%), very low (15%), and high (2%). For narrative syntheses, confidence in the reported outcomes was rated as moderate (36%), low (34%), or very low (30%). A detailed summary of the GRADE assessments is provided in **Figures S3** and **S4**.

#### Findings of Mediterranean DP on Inflammation and Strength of Evidence

The relationship between a Mediterranean DP and inflammation was investigated by random-effects meta-analyses of 27 experimental trials, mostly RCTs (*n* = 21) and observational studies (*n* = 1) conducted across 8 included reviews^23^^,^^66^^,^^67^^,^^70^^,^^79^^,^^89–91^ (**Table S5**). In addition, 29 narrative syntheses of intervention trials (*n* = 18) and observational studies (*n* = 11) across 10 reviews^65^^,^^66^^,^^68^^,^^69^^,^^81^^,^^83–86^^,^^91^ were included. **Figure S5** provides a complete report of the direction of effect/association for individual review syntheses.

##### CRP

The effect of diet on CRP levels was evaluated in 8 meta-analyses of intervention trials. Five of these reported significant reductions in CRP levels, ranging from -0.37 to -1.04 mg/L.^23^^,^^67^^,^^79^^,^^90^ Additionally a meta-analysis of observational studies demonstrated a significant reduction in CRP associated with Mediterranean DP adherence. The CoE for these findings varied from high to low (**Figures 4** and **5**). Similarly, narrative syntheses of CRP demonstrated predominantly favorable outcomes. In intervention trials, 67% (*n* = 4) reported beneficial effects, while 80% (*n* = 4) of observational studies showed beneficial associations. However, the CoE ranged from moderate to very low.

##### IL-6

Three meta-analyses of intervention trials consistently reported significant reductions in IL-6, ranging from -0.38 to -1.07 pg/mL, with moderate to low CoE (**Table S5**).^23^^,^^70^^,^^79^ However, narrative syntheses of intervention trials and observational studies yielded conflicting results (**Figure 7**).

##### TNF-α and E-selectin

Each proinflammatory biomarker was evaluated in 3 meta-analyses of intervention trials. A single meta-analysis demonstrated significant reductions in TNF-α levels, with very low CoE.^79^ However, the remaining meta-analyses found no significant effect on either TNF-α or E-selectin (moderate to low CoE).^23^^,^^67^^,^^70^^,^^79^ Narrative syntheses of intervention trials indicated no overall effect. In contrast, observational studies consistently reported a beneficial association, with very low CoE.

##### Other proinflammatory biomarkers

Intracellular adhesion molecule 1 (ICAM-1) was evaluated in 2 meta-analyses, consistently demonstrating significant reductions,^23^^,^^79^ while single meta-analyses reported significant reductions in IL-1ß^70^ and P-selectin and vascular cell adhesion molecule 1 (VCAM-1).^79^ Meta-analyses of the remaining proinflammatory markers, IL-8 and interferon-gamma (IFN-γ), reported no significant reductions^70^ (**Table S5**). Similarly, there was no overall beneficial effect/association revealed in narrative syntheses (**Figure 7**).

##### Adiponectin

The Mediterranean DP demonstrated a significant increase in adiponectin, an anti-inflammatory biomarker, in 2 out of 3 meta-analyses of intervention trials. The observed increases ranged from 0.59 to 1.69 µg/mL (low CoE) (**Figure 6**). Across all included reviews, no studies reported harmful effects of the Mediterranean DP on inflammation.

#### Findings of a Vegetarian DP on Inflammation and Strength of Evidence

The relationship between a vegetarian DP and inflammation was investigated by 9 random-effects pooled analyses of observational studies (*n* = 8) and intervention trials (*n* = 1), conducted across 4 included reviews^36^^,^^70^^,^^71^^,^^78^ (**Figures 4** and **5**). Additionally, narrative syntheses of observational studies (*n* = 9) and intervention trials (*n* = 4) across 4 reviews^36^^,^^65^^,^^69^^,^^71^ were included (**Figure 7**).

##### CRP

Meta-analyses of observational studies demonstrated inconsistent findings, with half reporting significant reductions in CRP levels, ranging from -0.61 to -3.91 mg/mL^36^^,^^71^ (low to very low CoE) (**Figure 4**). Notably, Menzel et al^71^ conducted separate meta-analyses of observational studies, based on patient health status, and reported a significant reduction (MD: -3.91 mg/mL; 95% CI: -5.23, -2.60) in CRP levels only in patients with impaired kidney function (**Figure 4**). Additionally, a single review performed subgroup analyses based on the minimum duration of vegetarianism and reported no association with CRP levels (very low CoE)^78^ (**Figure 5**). A single pooled analysis of RCTs found no effect on CRP levels.^70^ Narrative syntheses (*n* = 2) revealed inconsistent results, with interventional trials demonstrating a beneficial effect whereas observational studies indicated a direction of no association^70^^,^^78^ (**Figure 7**).

##### Fibrinogen

A meta-analysis of observational studies reported a significant reduction in fibrinogen levels (MD: -0.22 g/L; 95% CI: -0.41 to -0.04) with very low CoE^36^ (**Table S5**).

##### IL-6

A meta-analysis of observational studies reported a significant increase in IL-6 levels (very low CoE) (**Figure 5**)^78^; however, a sensitivity analysis (leave-one-out) found no positive association between IL-6 levels and long-term adherence to a vegetarian DP (>2 years).^78^ Narrative syntheses of intervention and observational studies revealed no consistent effect/association between the vegetarian DP and IL-6 or other proinflammatory markers (**Figure 7**).

##### Adiponectin

A single synthesis of observational studies revealed a beneficial association with anti-inflammatory adiponectin (low CoE) (**Figure 7**).

#### Findings of a Vegan DP on Inflammation and Strength of Evidence

The relationship between a vegan DP and inflammation was investigated by random-effects meta-analyses of observational studies (*n* = 2), conducted across 2 included reviews^36^^,^^71^ (**Figure 4**). Additionally, 4 narrative syntheses across 3 reviews^65^^,^^71^^,^^86^ were included (**Figure S5**).

##### CRP

Two meta-analyses of observational studies examining CRP levels yielded inconsistent findings: one showed a significant reduction (low CoE),^71^ whereas the other found no effect (very low CoE^36^) (**Figure 4**). A single narrative synthesis of intervention trials revealed a beneficial effect on CRP levels, with very low CoE (**Figure 7**).

##### Other inflammatory biomarkers

Narrative syntheses that assessed all other inflammatory markers, including adiponectin, revealed no effect/association (**Figure 7**).

Across all reviews, there were no reports of a vegan DP demonstrating harmful effects on inflammatory markers.

#### Findings of a Low-Glycemic DP on Inflammation and Strength of Evidence

The relationship between an LG DP and inflammation was investigated using random-effects (*n* = 2) and fixed-effects (*n* = 3) meta-analyses of RCTs, conducted across 3 included reviews^15^^,^^73^^,^^82^ (**Figures 4** and **6**). Six narrative syntheses across 3 reviews^83–85^ were included (**Figure 7**).

##### CRP

Three meta-analyses of RCTs assessed CRP levels, of which 2 reported significant reductions ranging from -0.41^73^ to -0.43 mg/L^15^ (moderate to low CoE) (**Figures 4** and **5**).

##### Other inflammatory biomarkers

Proinflammatory IL-6 and anti-inflammatory adiponectin were each assessed in a single meta-analyses of RCTs (**Figures 4** and **6**). An LG DP was associated with a significant amelioration of both markers, with moderate CoE. Conversely, narrative syntheses revealed no overall association with IL-6 or TNF-α concentrations (**Figure 7**). Across all reviews, there were no reports of an LG DP demonstrating a harmful effect on inflammatory markers.

#### Findings of Ketogenic DPs on Inflammation and Strength of Evidence

The relationship between a ketogenic DP and inflammation was investigated by random-effects meta-analyses of RCTs (*n* = 6), conducted across 2 included reviews^80^^,^^88^ (**Figure 4**). Meta-analyses of RCTs evaluated the effect of a ketogenic diet on CRP (*n* = 2), yielding inconsistent findings, and found effects on other proinflammatory markers, IL-6, IL-8, and TNF-α.^80^^,^^88^ The CoE ranged from very low to moderate (**Figure 4**).

#### Findings of Other DPs on Inflammation and Strength of Evidence

The association between the Nordic DP and inflammation was investigated in meta-analyses of RCTs and demonstrated no effect on CRP (*n* = 3), IL-6 (*n* = 1), or TNF-α (*n* = 1), with moderate CoE (**Figure 4**). The DASH DP was investigated in meta-analyses of RCTs (*n* = 2) and yielded mixed results for CRP, with 1 meta-analysis reporting a significant reduction (MD: -0.74 mg/mL; 95% CI: -1.36 to -0.11) with moderate CoE.^77^ However, a second meta-analysis^70^ and a single narrative review of observational studies^83^ found no relationship with CRP levels. The Portfolio^74^ and low-fat^72^ DPs were each evaluated in individual meta-analyses of intervention studies, demonstrating significant reductions in CRP levels. Lastly a narrative synthesis of observational studies revealed a beneficial association between the Paleolithic DP and CRP levels, and a harmful association with the Western DP^83^ (**Figure S5**).

### Heterogeneity and Publication Bias

Considerable heterogeneity was observed for outcomes meta-analyzed across most DPs, with authors of the reviews citing variability in participant characteristics, study design and duration,^23^^,^^70^^,^^71^^,^^80^^,^^88^^,^^91^ DP composition and/or definitions,^23^^,^^89^ inconsistencies in methods used to measure dietary adherence,^70^^,^^79^^,^^80^^,^^91^ differences in inflammatory marker selection and assay quality measurements,^71^ limited studies and small sample sizes,^23^^,^^66^^,^^91^^,^^102^ and broad variability in mean baseline CRP values across studies.^66^

The majority of meta-analyses, with 10 or more comparisons, performed publication bias analyses using the Begg's and Egger's tests^79^^,^^80^^,^^103^ and reported no evidence of substantial publication bias or small study effects.^15^^,^^23^^,^^36^^,^^67^^,^^70^^,^^71^^,^^76–78^^,^^80^^,^^89–91^ Three reviews did not report on publication bias analyses.^66^^,^^82^^,^^88^ Last, due to the small number of studies (<10) that investigated inflammatory marker outcomes, several reviews were unable to assess publication bias.^60^^,^^72–75^^,^^77^^,^^78^^,^^89^

## DISCUSSION

This umbrella review presents a comprehensive and critical assessment of the existing body of evidence regarding the relationship between DPs and inflammation. Of the 30 reviews, representing 225 unique primary studies, 15 DPs were assessed against a range of inflammatory marker outcomes reported in 60 pooled analyses (individual meta-analyses), with moderate (52%), low (30%), very low (15%), and high (2%) CoE, and 61 narrative syntheses with moderate (36%), low (34%), and very low (30%) CoE.

Findings from this umbrella review demonstrate a significant inverse effect and overall beneficial association between the Mediterranean DP and low-grade inflammation, as indicated by CRP, IL-6, and adiponectin levels, in adult populations with at least 1 chronic condition. These findings can only be considered in the context of the broad variation in CoE, extending from high to low, and a number of deficiencies in the evidence indicate that additional research is required to determine whether the estimated effect is the true effect. Additionally, the review found low to very low levels of CoE for the beneficial association between the vegetarian DP and CRP levels, implying that it is likely that the body of evidence has major or unacceptable deficiencies. Further research is required to determine whether these findings are robust and accurately reflect the true effect/association.

The Mediterranean DP is the most extensively examined DP in nutrition research.^26^^,^^92^ As described, this pattern typically includes a high consumption of vegetables, fruits, whole grains, legumes, nuts, and olive oil, with moderate intakes of fish, poultry, and red wine, and limited consumption of red meat and processed foods.^25^^,^^26^^,^^104–106^ Large observational studies and intervention trials have previously provided strong evidence for the inflammation-modulating effects of the Mediterranean DP, leading to lower concentrations of CRP (20%), IL-6 (17%), and fibrinogen (6%).^21^^,^^107^ The substantial evidence supporting the use of the Mediterranean DP for secondary prevention of CVD has been attributed to its beneficial effects on chronic inflammation.^26^^,^^92^ Lower-inflammatory diets have been associated with a substantial risk reduction (hazard ratio: 0.61; 95% CI: 0.60–0.63) of incident major CVD, T2D, and certain cancers.^17^ A recent, comprehensive analysis of 116 longitudinal studies revealed the principal components associated with healthy DPs including vegetables, fruits, fish and seafood, whole grains, low-fat dairy products, poultry, soya, legumes, olive oil, nuts and seeds, and beans. The most common foods contributing to unhealthy DPs included red and processed meat, refined grains, high-fat dairy products, sweets, cakes, and biscuits.^108^ The Mediterranean, DASH, Nordic, and other plant-based DPs show considerable concurrence, all emphasizing foods associated with lower inflammation, including whole grains and legumes, vegetables, and fruits (specifically berries in the case of the Nordic DP), nuts, and fish.^19^^,^^109^^,^^110^ Similarly, the Portfolio DP emphasizes intake of nuts, soy protein, and foods rich in viscous fiber and is associated with significantly lower inflammatory markers.^100^^,^^111^

The beneficial health effects of dietary components associated with plant-based DPs have been widely researched,^112^ although the extent of their anti-inflammatory effects remains less certain.^19^^,^^78^ The exact anti-inflammatory mechanisms of these dietary components are yet to be fully elucidated, and outcomes vary depending on dietary composition and duration of adherence.^113^^,^^114^ Nevertheless, researchers have attributed the anti-inflammatory effects of the Mediterranean and vegetarian DPs to several key biological mechanisms. The monounsaturated and omega-3 polyunsaturated fatty acids, present in fish and nuts, inhibit the production of proinflammatory biomarkers and regulate the expression of inflammatory genes.^115^ The high-fiber content of these diets promotes gut health, fostering a diverse and beneficial microbiota that produces short-chain fatty acids (SCFAs), such as butyrate. SCFAs maintain intestinal barrier integrity and inhibit inflammation, by regulating immune responses.^116^^,^^117^ Fiber further ameliorates systemic inflammation by binding to toxins and bile acids, thereby reducing their absorption into the bloodstream.^118^ Plant-based diets are characterized by an abundance of foods rich in polyphenol content, such as fruits, vegetables, legumes, nuts, and olive oil.^92^ Polyphenols are potent antioxidants that exert anti-inflammatory effects by inhibiting the activation of proinflammatory pathways, and reducing oxidative stress.^119^ Although several biological mechanisms support the anti-inflammatory potential of these diets, the evidence regarding other DPs, such as the Paleolithic DP, remains inconclusive and warrants careful interpretation. In a recent network meta-analysis by Liang et al^112^ the Paleolithic DP ranked highest for an overall beneficial effect on NCD biomarker outcomes, including CRP. However, the current umbrella review suggests that the beneficial effect observed for the Paleolithic DP on CRP should be interpreted with caution, due to the very low CoE from a single synthesis of observational studies.^69^

Evidence on the effects of several reviewed DPs on chronic inflammation remains inconclusive, and it is important to consider why our results are limited given the amount of evidence synthesized. For instance, the lack of definitive findings may be attributed to the inherent limitations of restrictive diets, such as vegan and ketogenic DPs, which are often defined by what they exclude rather than their overall composition.^120^^,^^121^ This fails to account for variations in food quality, micronutrient content, and dietary diversity.^122^^,^^123^ This heterogeneity complicates the attribution of inflammatory marker changes to specific DPs. Indeed, the observed inconsistencies in the findings may be related to the high heterogeneity and broad variability noted in study design, participant population, comparator diet, and limited range of inflammatory biomarkers measured.^19^ Nutrition and the examination of DPs is a complex field of research, subject to numerous challenges that are inherent to this area of inquiry.^124^ Researchers are frequently required to make subjective judgements that may affect the study outcomes^125^—for instance, deciding the number of food groups and components entered into DP analysis, and the selection of DPs included in analyses with health outcomes.^125^^,^^126^ Furthermore, obtaining accurate dietary data from participants remains challenging.^127–129^ Commonly, and relevant to the present review, dietary data are collected through self-reported methods, including FFQs, 24HRs, and 3-day food records.^127^ These methods are limited by several biases, including mis- or underreporting, which may vary depending on personal traits, including gender, body weight, or inability to accurately estimate food intake.^127^^,^^130^^,^^131^ As a point for future research, the intra-variability in 24HRs and the specific time periods covered by FFQs are important considerations that should be carefully evaluated to ensure comprehensive dietary assessment.^127^ Biomarkers have also been explored as a means of assessing food consumption, but they come with their own complications, such as variations in nutrient absorption among individuals.^132^ They may also only represent recent intake, and this is not necessarily helpful in longitudinal studies. Last, in order for evidence from DPs to be effectively synthesized and translated into practice, it is important that the methods used to examine whole dietary patterns are adequately described.^125^ In the current review, there was a distinct lack of detail in the reporting of dietary intake assessments and methods used to construct DPs. Additionally, the level of detail used to define the DPs was inconsistent and, in many instances, was not documented.

An additional conundrum in this field of research is the lack of consensus on what constitutes a clinically meaningful reduction in the concentrations of inflammatory biomarkers. Nonetheless, there is evidence to suggest that CRP is independently associated with mortality, and elevated levels of CRP (≥10 mg/L) can serve as a useful predictor of adverse cardiovascular events and all-cause mortality over an 8-year period.^133^ For each 1-mg/L increase in serum CRP concentration there has been determined to be an 8% increase in recurrent CVD risk.^134^ Therefore in the current review, the observed significant reductions in CRP concentrations ranging from -0.26^91^ to -1.04 mg/L^79^ in relation to the Mediterranean DP and -0.25 to 3.91 mg/mL^71^ for the vegetarian DP may be highly relevant to outcomes. However, traditional inflammatory markers, such as CRP, IL-6, and TNF-α, are sensitive to numerous factors, including age, adiposity, sex, genetics, gut microbiome composition, smoking status, physical inactivity, medication use, stress, and environmental pollutants.^10^^,^^114^ Therefore, studies must control for these factors when possible.

Furthermore, while these markers provide valuable insight into the relationship between inflammatory activity and disease and mortality risk, they offer limited mechanistic information. Given the enormous complexity of the inflammatory response, they may not fully capture the nuances of low-grade inflammatory processes or regulatory pathways observed in inflammation-related chronic diseases.^10^ Future research should thus include novel inflammatory biomarkers that may provide a more direct link to specific inflammatory pathways, potentially providing deeper insights into chronic inflammation.^65^ Notably, the current review included only a single systematic review investigating DPs and novel inflammatory markers.^65^

A more comprehensive and holistic approach to evaluating inflammatory status may be warranted.^114^ Nutritional interventions should target patterns or clusters of inflammatory markers, as they may provide more reliable indicators of chronic inflammation than individual markers alone. Clusters of markers identified in longitudinal studies have been linked to mortality and frailty, lending these markers potential clinical significance.^28^^,^^114^ However, despite the emergence of new techniques, they have yet to be extensively validated for clinical applications.^122^^,^^135^ Although they show promise, these marker clusters require further study and refinement to address their limitations and increase applicability across populations and disease states.^65^^,^^114^

An additional key factor that may have contributed to the limited definitive outcomes here relates to comparator diets or DPs. To comprehensively capture the evidence, no limitation was placed on comparator diets in the primary studies. This led to a high degree of variability and heterogeneity across meta-analyses and narrative syntheses. Gardner et al^136^ asserted that “instead of what” and “in what context” are important factors that should be consistently addressed in nutrition research. Significant heterogeneity highlights the need for further primary studies to consider the effects of DPs according to delineated comparator diets and future meta-analyses to calculate anti-inflammatory effects based on healthy DPs even within the same intervention. Mixing and combining comparator diets is a limitation in measuring outcomes.

Last, it is noteworthy that, while the Mediterranean DP demonstrated the most well-characterized benefits, studies indicate that adherence to this diet is notably low, particularly among young people in North America, Oceania, and most parts of Europe.^137–139^ For example, fruit and vegetable intake is substantially below recommendations for a Mediterranean DP. This is concerning, given that low fruit, vegetable, and whole-grain consumption are among the leading dietary risk factors for death and morbidity globally.^140^^,^^141^ Suboptimal adherence to healthy DPs among adolescents may lead to significant adverse health outcomes in adulthood, potentially contributing to the high prevalence of chronic diseases observed in these regions.^139^^,^^142^ The global adoption of a Mediterranean DP seems improbable, and even in countries where the Mediterranean DP originated, there is a concerning trend away from healthful eating habits.^142^ Healthy dietary patterns are characterized by both nutrient adequacy and the avoidance of excessive consumption of foods associated with increased health risks.^142–145^ The shift from traditional DPs, based on minimally processed plant foods, to diets high in red and processed meats and ultra-processed products high in added sugars, fats, and sodium is part of a larger nutrition transition occurring globally, particularly in low- and middle-income countries.^140^^,^^146^ Therefore, if we are to promote beneficial DPs, it is crucial to consider the multifactorial influences on food choices, including food supply, food literacy, socioeconomic status, food trends, and culture.^140^^,^^142^^,^^143^^,^^147^ Implementing the adoption of and increased adherence to such DPs requires nutritional education and behavior change strategies that specifically target common barriers, including time constraints, limited cooking skills, food access issues, and ingrained eating habits.^147^^,^^148^ Nonetheless, the beneficial health effects of plant-based DPs, as reviewed in this synthesis, and the consistency in dietary components that exists between these DPs highlight the opportunities for contextualization of the diets to the local food supply.^112^^,^^149^ Public health strategies should align dietary recommendations with local food supplies and cultural practices, tailoring plant-based patterns to regional contexts while preserving beneficial components. This approach enhances the relevance, feasibility, acceptability, and sustainability of beneficial DPs across diverse populations.^148^^,^^150^

### Strengths and Limitations

The umbrella review incorporates the highest level of evidence, providing a comprehensive summary and evaluation of the existing evidence.^32^^,^^149^^,^^151^ The current review is strengthened by an adherence to established methodological guidelines and standardized procedures (preregistered protocol, Cochrane Handbook^32^) Additionally, validated assessment tools (AMSTAR-2,^38^ GRADE algorithm^46^) were applied to reduce noise and uncertainty in interpreting and applying the findings. The authors robustly assessed the extent of overlap (CCA^40^) and determined this to be slight (1.6%). Additionally, the pooling of effect sizes is not typically re-analyzed in umbrella reviews, which further minimizes the risk of overlap between studies.^32^^,^^151^

There are several limitations inherent to the umbrella review methodology. First, the umbrella review is dependent on the accuracy and rigor of the methodological quality of the published systematic reviews and meta-analyses, as well as the primary studies they include.^151^ Notably, the methodological quality, assessed using the AMSTAR-2 critical appraisal, was low/critically low for one-third of the included reviews and may compromise the representativeness of the findings.^151^ However, in an umbrella review, studies are not excluded based on methodological quality, as this would result in a loss of information.^39^ Second, publication bias may affect the overall findings of the umbrella review,^151^ and due to a lack of sufficient comparisons for inflammatory outcomes, publication bias was not assessed in several of the included analyses. However, most reviews with sufficient data performed publication bias assessments and found no evidence of bias, adding to the completeness of the reported results. Third, the umbrella review only considers evidence from systematic reviews and meta-analyses, thereby excluding the latest evidence from studies not yet included in these reviews.^151^ Last, the current umbrella review was restricted to studies published in English. Consequently, relevant studies published in other languages may have been missed, potentially compromising the comprehensiveness of the findings and introducing “English-language bias.”^152^

Despite these limitations, this thorough examination of 15 distinct DPs provides a comprehensive understanding of the evidence regarding DPs and their effect on inflammation. Moreover, the incorporation of reviews encompassing a broad range of health conditions and populations adds depth to the current review findings.

### Future Research

Future research should focus on the utilization of biomarkers to better understand the biological mechanisms and differences in inflammation among various DPs.^36^^,^^78^^,^^83^^,^^90^ This includes the use of biological markers of dietary intake in addition to self-reported data.^69^^,^^83^ The field should also explore additional novel inflammatory markers, including validation studies to establish reference values in diverse populations.^15^^,^^65^ Large-scale, adequately powered intervention trials with longer durations, such as those lasting over 12 months, should be conducted to determine the effects of various healthy and/or isocaloric DPs on novel inflammatory markers and assess their clinical utility in different populations.^65^^,^^67^^,^^69–72^^,^^82^^,^^89^ Finally, utilizing standardized definitions of DPs, further primary research and meta-analyses are needed to evaluate the effects of DPs relative to specific comparator diets.

## CONCLUSION

This umbrella review assessed all available systematic reviews and meta-analyses of the association between DPs and inflammation. The findings underscore the surprisingly limited number of available studies on some DPs and further highlight the complexity and heterogeneity inherent in studying DPs. Despite this, the results suggest that, in adult populations with at least 1 chronic condition, the Mediterranean DP, characterized by a high consumption of fruits, vegetables, whole grains, legumes, and healthy fats, demonstrated significant anti-inflammatory effects across meta-analyses and narrative syntheses, with high to low level CoE. Similarly, although with very low to low CoE, the vegetarian DP exhibited anti-inflammatory effects, particularly in reducing CRP levels. Future studies are needed to comprehensively categorize the effects of DPs according to delineated comparator diets, emphasizing the importance of consistently addressing the “instead of what” factor in nutrition research.

## Supplementary Material

nuaf104_Supplementary_Data

## Data Availability

All data described and presented in the article will be made available upon request.
