# Supplementary material for: Dietary Patterns Associated With Anti-inflammatory Effects: An Umbrella Review of Systematic Reviews and Meta-analyses
Source: Nutr Rev. 2025 Jul 14;84(6):1167–92. doi: 10.1093/nutrit/nuaf104 (PMC13161761; doi:10.1093/nutrit/nuaf104)
Supplement: nuaf104_Supplementary_Data [file nuaf104_supplementary_data.zip › Manuscript Supplemental Material.docx]

**Dietary patterns associated with anti-inflammatory effects: an umbrella review of systematic reviews and meta-analyses.**

Gynette L. Reyneke^1^, Kelly Lambert^2^, Eleanor J. Beck^1,2^

^1^School of Health Sciences, University of New South Wales, Sydney, New South Wales, Australia.

^2^School of Medical, Indigenous, and Health Sciences, University of Wollongong, Wollongong, New South Wales, Australia.

**Corresponding author:**

Eleanor Beck

Address: University of New South Wales, Sydney, New South Wales Australia 2052

Email: e.beck@unsw.edu.au

Online Supplemental Material

## Supplemental Tables

### **Supplemental Table S1.** Preferred Reporting Items for Overviews of Reviews (PRIOR)

| Selection  Topic | # | Item | Location reported |  |
| --- | --- | --- | --- | --- |
|  |  |  | Page | Line |
| **TITLE** | | | |  |
| Title | 1 | Identify the report as an overview of reviews. | 1 | 1-2 |
| **ABSTRACT** | | | |  |
| Abstract | 2 | Provide a comprehensive and accurate summary of the purpose, methods, and results of the overview of reviews. | 3 | 50-72 |
| **INTRODUCTION** | | | |  |
| Rationale | 3 | Describe the rationale for conducting the overview of reviews in the context of existing knowledge | 4-5 | 75-118 |
| Objectives | 4 | Provide an explicit statement of the objective(s) or question(s) addressed by the overview of reviews. | 5 | 118-124 |
| **METHODS** | | | |  |
| Eligibility criteria | 5a | Specify the inclusion and exclusion criteria for the overview of reviews. If supplemental primary studies were included, this should be stated, with a rationale. | 6  37 | 148-155  Table 1 |
|  | 5b | Specify the definition of ‘systematic review’ as used in the inclusion criteria for the overview of reviews. | 6  37 | 153-155  Table 1 |
| Information sources | 6 | Specify all databases, registers, websites, organizations, reference lists, and other sources searched or consulted to identify systematic reviews and supplemental primary studies (if included). Specify the date when each source was last searched or consulted. | 6 | 138-145 |
| Search strategy | 7 | Present the full search strategies for all databases, registers and websites, such that they could be reproduced. Describe any search filters and limits applied. | 6 | 140-144 |
| Selection process | 8a | Describe the methods used to decide whether a systematic review or supplemental primary study (if included) met the inclusion criteria of the overview of reviews | 7 | 160-175 |
|  | 8b | Describe how overlap in the populations, interventions, comparators, and/or outcomes of systematic reviews was identified and managed during study selection | 8 | 194-204 |
| Data collection process | 9a | Describe the methods used to collect data from reports. | 7-8 | 178-191 |
|  | 9b | If applicable, describe the methods used to identify and manage primary study overlap at the level of the comparison and outcome during data collection. For each outcome, specify the method used to illustrate and/or quantify the degree of primary study overlap across systematic reviews. | 8 | 185-195 |
|  | 9c | If applicable, specify the methods used to manage discrepant data across systematic reviews during data collection. | 7 | 174-175 |
| Data items | 10 | List and define all variables and outcomes for which data were sought. Describe any assumptions made and/or measures taken to identify and clarify missing or unclear information. | 8 | 181-185 |
| Risk of bias assessment | 11a | Describe the methods used to assess risk of bias or methodological quality of the included systematic reviews. | 8-9 | 207-223 |
|  | 11b | Describe the methods used to collect data on (from the systematic reviews) and/or assess the risk of bias of the primary studies included in the systematic reviews. Provide a justification for instances where flawed, incomplete, or missing assessments are identified but not re-assessed. | 9 | 207-209 |
|  | 11c | Describe the methods used to assess the risk of bias of supplemental primary studies (if included). | N/A | N/A |
| Synthesis methods | 12a | Describe the methods used to summarize or synthesize results and provide a rationale for the choice(s). | 9-10 | 226-240 |
|  | 12b | Describe any methods used to explore possible causes of heterogeneity among results. | N/A | N/A |
|  | 12c | Describe any sensitivity analyses conducted to assess the robustness of the synthesized results | N/A | N/A |
| Reporting bias assessment | 13 | Describe the methods used to collect data on (from the systematic reviews) and/or assess the risk of bias due to missing results in a summary or synthesis (arising from reporting biases at the levels of the systematic reviews, primary studies, and supplemental primary studies, if included). | 9 | 207-213 |
| Certainty assessment | 14 | Describe the methods used to collect data on (from the systematic reviews) and/or assess certainty (or confidence) in the body of evidence for an outcome. | 10 | 243-254 |
| **RESULTS** | | | |  |
| Systematic review and supplemental primary study selection | 15a | Describe the results of the search and selection process, including the number of records screened, assessed for eligibility, and included in the overview of reviews, ideally with a flow diagram | 10-11 | 257-269  Figure 1 |
|  | 15b | Provide a list of studies that might appear to meet the inclusion criteria, but were excluded, with the main reason for exclusion. | Supplemental Material | Supplemental Table 3 |
| Characteristics of systematic reviews and supplemental primary studies | 16 | Cite each included systematic review and supplemental primary study (if included) and present its characteristics. | 11-13 | 272-303; 323-335  Table 3 |
| Primary study overlap | 17 | Describe the extent of primary study overlap across the included systematic reviews. | 14 | 338-343  Figure 3 |
| Risk of bias in systematic reviews, primary studies, and supplemental primary studies | 18a | Present assessments of risk of bias or methodological quality for each included systematic review | 14 | 346-353  Supplemental Figure 1-2 |
|  | 18b | Present assessments (collected from systematic reviews or assessed anew) of the risk of bias of the primary studies included in the systematic reviews | Supplemental Material | Supplemental Figure 1-2 |
|  | 18c | Present assessments of the risk of bias of supplemental primary studies (if included). | N/A |  |
| Summary or synthesis of results | 19a | For all outcomes, summarize the evidence from the systematic reviews and supplemental primary studies (if included). If meta-analyses were done, present for each the summary estimate and its precision and measures of statistical heterogeneity. If comparing groups, describe the direction of the effect. | 13-18 | 356-493  Figures 4-7  Supplemental Table S5 and Supplemental Figure S5 |
|  | 19b | If meta-analyses were done, present results of all investigations of possible causes of heterogeneity. | N/A | N/A |
|  | 19c | If meta-analyses were done, present results of all sensitivity analyses conducted to assess the robustness of synthesized results. | N/A | N/A |
| Reporting biases | 20 | Present assessments (collected from systematic reviews and/or assessed anew) of the risk of bias due to missing primary studies, analyses, or results in a summary or synthesis (arising from reporting biases at the levels of the systematic reviews, primary studies, and supplemental primary studies, if included) for each summary or synthesis assessed. | 18 | 496-508 |
| Certainty of evidence | 21 | Present assessments (collected or assessed anew) of certainty (or confidence) in the body of evidence for each outcome | Supplemental Material | Supplemental Figures S3 and S4 |
| **DISCUSSION** | | | |  |
| Discussion | 22a | Summarize the main findings, including any discrepancies in findings across the included systematic reviews and supplemental primary studies (if included). | 19-20 | 511-715 |
|  | 22b | Provide a general interpretation of the results in the context of other evidence. | 20-24 | 518-681 |
|  | 22c | Discuss any limitations of the evidence from systematic reviews, their primary studies, and supplemental primary studies (if included) included in the overview of reviews. Discuss any limitations of the overview of reviews methods used. | 25-26 | 684-715 |
|  | 22d | Discuss implications for practice, policy, and future research (both systematic reviews and primary research). Consider the relevance of the findings to the end users of the overview of reviews, e.g., healthcare providers, policymakers, patients, among others. | 27 | 718-724 |
| **OTHER INFORMATION** | | | |  |
| Registration and protocol | 23a | Provide registration information for the overview of reviews, including register name and registration number, or state that the overview of reviews was not registered. | 6 | 132-134 |
|  | 23b | Indicate where the overview of reviews protocol can be accessed, or state that a protocol was not prepared. | 6 | 134 |
|  | 23c | Describe and explain any amendments to information provided at registration or in the protocol. Indicate the stage of the overview of reviews at which amendments were made | N/A | N/A |
| Support | 24 | Describe sources of financial or non-financial support for the overview of reviews, and the role of the funders or sponsors in the overview of reviews. | 28 | 754 |
| Competing interests | 25 | Declare any competing interests of the overview of reviews' authors. | 28 | 756 |
| Author information | 26a | Provide contact information for the corresponding author. | 1 | 12-14 |
|  | 26b | Describe the contributions of individual authors and identify the guarantor of the overview of reviews. | 28 | 744-749 |
| Availability of data and other materials | 27 | Report which of the following are available, where they can be found, and under which conditions they may be accessed: template data collection forms; data collected from included systematic reviews and supplemental primary studies; analytic code; any other materials used in the overview of reviews. | 28 | 751-752 |

**Supplemental Table S2****.** Search Strategy for individual databases (19/03/2025)

| **NO.** | **SEARCH FIELD** | **LIMITERS - ENGLISH LANGUAGE; HUMAN**  **SEARCH MODES - BOOLEAN/PHRASE** |
| --- | --- | --- |
| CINAHL database (n = 571) | | |
| *Title, Abstract, Subject,*  *MeSH Term* | Diet OR diets OR "dietary pattern*" OR "eating pattern*" OR diet+ OR diet therapy+ [MeSH Term] AND inflammation OR inflammatory OR anti-inflammation OR anti-inflammatory OR "anti inflammation" OR "anti inflammatory" OR inflammation [MeSH Term] OR inflammation mediators+ [MeSH Term] AND "systematic review" OR "meta-analysis" OR "meta analysis" OR "systematic literature review" OR systematic review [MeSH Term] OR meta-analysis [MeSH Term] | |
| Pubmed database (n = 954) | | |
| *Title/ Abstract, Text Word, MeSH Terms* | Diet OR diets OR "dietary pattern*" OR "eating pattern*" OR diet [MeSH Term] OR “diet therapy” [MeSH Term] AND inflammation OR inflammatory OR anti-inflammation OR anti-inflammatory OR "anti inflammation" OR "anti inflammatory" OR "inflammation mediators” [MeSH Term] AND "systematic review" OR "meta-analysis" OR "meta analysis" OR "systematic literature review" OR systematic review [MeSH Term] OR meta-analysis [Text Word] | |
| SCOPUS database (n = 556) | | |
| *Title/ Abstract, Keywords* | Diet OR diets OR "dietary pattern*" OR "eating pattern*" OR “diet therapy” AND inflammation OR inflammatory OR anti-inflammation OR anti-inflammatory OR "anti inflammation" OR "anti inflammatory" OR "inflammation mediators" AND "systematic review" OR "meta-analysis" OR "meta analysis" OR "systematic literature review" | |
| Web of Science Core Collection database (n = 1033) | | |
| *Title, Abstract, Keywords*  *Plus* | Diet OR diets OR "dietary pattern*" OR "eating pattern*" OR diet therapy AND inflammation OR inflammatory OR anti-inflammation OR anti-inflammatory OR "anti inflammation" OR "anti inflammatory" OR "inflammation mediators" AND "systematic review" OR "meta-analysis" OR "meta analysis" OR "systematic literature review" | |
| Cochrane database (n = 163) | | |
| *Title, Abstract, Keyword*  *and MeSH Term* | Diet OR diets OR "dietary pattern" OR "dietary patterns" OR "eating pattern" OR "eating patterns" OR “diet therapy” OR “diet” [MeSH Term] AND inflammation OR inflammatory OR anti-inflammation OR anti-inflammatory OR "anti inflammation" OR "anti inflammatory" OR "inflammation mediators" AND "systematic review" OR "meta-analysis" OR "meta analysis" OR "systematic literature review" or SYSTEMATIC REVIEW+ [MeSH Term] | |

### **Supplemental Table S3.** Full details of excluded reviews and primary studies

| **Review reference** | **Type** | **Total**  **Primary studies** | **Eligible primary studies** | **Reason/ details** |
| --- | --- | --- | --- | --- |
| Abdallah 2023 ^1^ | SR | 7 | Review Excluded | Full review excluded. A single primary study (PS) met eligibility criteria and therefore does not meet the criteria for a narrative synthesis. All other PS did not meet the eligibility criteria the current umbrella review: (i) did not assess the association between dietary interventions and measured outcomes of inflammatory markers (*n* = 5); eligible studies that assessed the effect of DASH DP on markers of inflammation had a 100% overlap with another review (*n* = 1) |
| Akbar 2023^2^ | SR | 23 | Review excluded | Full review excluded. A single primary study (PS) met eligibility criteria and therefore does not meet the criteria for a narrative synthesis. All other PS did not meet the eligibility criteria the current umbrella review: did not assess the association between dietary interventions and measured outcomes of inflammatory markers (*n* = 22). |
| Aleksandrova 2021 ^3^ | SR | 29 | 8 | Primary studies did not meet the eligibility criteria: (i) did not assess the association between dietary interventions and measured outcomes inflammatory markers (*n* = 17); (ii) assessed the effect of single foods/ food groups on inflammatory markers (*n = 2*); (iii) duration < 4 weeks (*n* = 2) |
| Apekey 2022 ^4^ | SRMA | 25 | Review excluded | Review excluded due to serious methodological issues regarding the meta-analyses conducted by the review authors (*n* = 8) (i) meta-analysis for IL-6 conducted using a single study. In accordance with the Cochrane collaboration, a minimum of two studies (trial comparisons) are required for a meta-analysis; (ii) meta-analysis for pooled effect of low carbohydrate DP on CRP includes data for mean difference (95% confidence interval) that differs substantially from the data reported in the primary study by Saslow et al., 2014. All other primary studies were not included in the review synthesis or meta-analysis (*n =* 17). |
| Bujtor 2021^5^ | SR | 53 | 18 | Primary studies did not meet the eligibility criteria: (i) assessed the effect of single foods/ food groups on inflammatory markers (*n =* 25); (ii) assessed the effect of single foods/ food groups on inflammatory markers (*n =* 9); (iii) assessed the effects of a combined intervention including Mediterranean diet, muscular and cardiorespiratory fitness on CRP levels, therefore unable to separate out the effects of the Mediterranean dietary pattern on hs-CRP (*n* = 1); |
| Chiavaroli 2021^6^ | SRMA | 27 | 6 | Primary studies did not meet the eligibility criteria: (i) did not assess the association between dietary interventions and measured outcomes of inflammatory markers (*n =* 21). |
| Cowan 2020^7^ | SR | 33 | Review excluded | Primary studies did not meet the eligibility criteria: (i) assessed the effect of single foods/ food groups on inflammatory markers (*n =* 31); (ii) duration < 4 weeks (*n* = 2) |
| Craddock 2019^8^ | SRMA | 40 | 30 | Primary studies did not meet the eligibility criteria: (i) measured outcomes did not include any inflammatory markers (*n* = 10); |
| De Sevilla 2022^9^ | SR | 5 | Review excluded | Primary studies did not meet the eligibility criteria: (i) did not assess the association between dietary interventions and measured outcomes inflammatory markers. |
| Dos Reis Padilha 2018^10^ | SR | 12 | Review Excluded | Full review excluded. A single primary study (PS) met eligibility criteria and therefore does not meet the criteria for a narrative synthesis. All other PS did not meet the eligibility criteria: (i) did not assess the association between dietary interventions and measured outcomes inflammatory markers (*n* = 11) |
| Eichelmann 2016^11^ | SRMA | 28 | Review excluded | The meta-analysis combined data from multiple DPs therefore effect of specific DP could not be separated out. Therefore, the review does not meet the inclusion criteria of the current umbrella review which seeks to determine the evidence for specific dietary patterns. The review did not include an additional narrative synthesis that could be included under the qualitative findings. |
| English 2022^12^ | SR | 16 | 6 | Primary studies did not meet the eligibility criteria: (i) assessed association between foods/ food groups and inflammatory biomarkers outcomes (*n* = 7); (ii) included pharmacological element and therefore unable to separate out the effects of dietary intervention (*n* = 1); (iii) included a dietary intervention + placebo + exercise regime, therefore unable to separate out the effects of dietary intervention (*n* = 1); (iv) study duration <4 weeks (*n* = 1) |
| Ghaedi 2019^13^ | SRMA | 8 | Review excluded | The meta-analyses included a primary study (PS) with a duration of <4 weeks and therefore was not included in the current umbrella review. Additionally, PS did not assess the association between dietary interventions and measured outcomes inflammatory markers (*n* = 3). The review did not include an additional narrative synthesis that could be included under the qualitative findings |
| Grammatikopoulou 2020^14^ | SR | 4 | 3 | Primary studies did not meet the eligibility criteria: (i) assessed association between food components and inflammatory biomarkers outcomes (*n* = 1) |
| Haghighatdoost 2017^15^ | SRMA | 18 | 17 | Review authors reported that primary study was not included in the meta-analyses because data were reported as median and interquartile range (*n* = 1) |
| Hart 2021^16^ | SR | 69 | Review excluded | The review results and synthesis lack sufficient information to determine (i) the sample sizes or the specific primary studies that contribute to the findings for each dietary pattern; (ii) the inclusion of primary studies that utilised indices/ scores that assess the effect of nutrients or single foods on inflammatory markers |
| Ilari 2023 ^17^ | SRMA | 65 | Review excluded | Review excluded due to serious methodological issues including:  (i) the authors did not identify the guidelines used to inform methodology for conducting the SRMA; (ii) completed PRISMA checklist was not included, therefore, unable to verify that all PRISMA items have been addressed in the study; (iii) critical appraisals to assess the quality of the included studies was not conducted; (iv) Figure 6. purports to illustrate the effects of the MED on IL-6 levels. This differs substantially from the data reported in the primary study by Yeo et al., 2011, which makes no mention of the MED. This inclusion raises concerns about the relevance and accuracy of the data presented; (v) Figure 9. purports to illustrate the effects of vegan diet on CRP and incorporates findings reported by Menzel et al., 2020. However, the reference provided for this publication is an SRMA and inclusion raises concerns about the relevance and accuracy of the data presented. |
| Ji 2025 | SRMA | 44 | 21 | Of the 44 RCTs included in the SRMA, several did not meet the specified duration of ≥4 weeks (n = 6). Consequently, any meta-analyses that incorporated these ineligible RCTs were excluded from the review. This resulted in the exclusion of an additional 17 RCTs from the review, as these were only reported as part of meta-analyses that included ineligible RCTs. In total, 23 RCTs were excluded from the review due to these criteria, ensuring that only studies reported in eligible meta-analyses were included in the final analysis. |
| Kazeminasab 2024 ^18^ | SRMA | 51 | Review excluded | Of the 51 RCTs included in the SRMA, several did not meet the specified duration of ≥4 weeks (n = 4). Consequently, any meta-analyses that incorporated these ineligible RCTs were excluded from the review. Authors conducted subgroup analyses by intervention duration for moderate-term interventions (12-24 weeks) and long-term interventions (>24 weeks) (supplementary material) however the specific RCTs incorporated in these subgroup analyses were not made available by the authors. The details of all studies included in meta-analyses is required for the current review in order to determine eligibility of the primary study, assess overlap and assess CoE. |
| Klonizakis 2021^19^ | SR | 20 | Review excluded | Review excluded due to 100% overlap of eligible primary studies. Eligible studies for Mediterranean and New Nordic DPs had 100% overlap with another review (n = 6). All other primary studies did not meet the eligibility criteria: (i) did not assess the association between dietary interventions and measured outcomes inflammatory markers (n = 11); (ii) assessed the association between single food components and inflammatory biomarkers (n = 2); (iii) included multiple lifestyle components including cooking classes, educational sessions and resources and therefore the findings for the effect of DP cannot be separated out (n = 1). |
| Koelman 2022^20^ |  | 23 | 22 | Review authors reported that primary study was not included in the meta-analyses due to insufficient data to calculate mean biomarker differences (*n* = 1) |
| Lari 2021^21^ |  | 54 | Review excluded | Review excluded due to 100% overlap of eligible primary studies. Eligible studies that assessed the effect of DASH DP on markers of inflammation had a 100% overlap with another review (*n* = 5); (ii) study duration <4 weeks (*n* = 1); All other primary studies did not assess the association between dietary interventions and measured outcomes inflammatory markers (*n* = 48) |
| Massara 2022^22^ |  | 21 | 5 | Primary studies did not meet the eligibility criteria: (i) findings were not included in the review synthesis or meta-analysis (*n =* 16). |
| Mayr 2018^23^ |  | 11 | 8 | Primary studies did not meet the eligibility criteria: (i) assessed the association between wine intake and inflammatory markers (n = 1); (ii) included an educational component and therefore the findings for the effect of DP cannot be separated out (n = 2). |
| Mukherjee 2022^24^ | SR | 20 | 14 | Review authors provided a narrative synthesis for 14 primary studies that assessed association between a Mediterranean DP and marker of inflammation. All other primary studies were not summarised in the narrative synthesis and included the DASH DP (*n* = 2), Portfolio DP (*n* =1) and anti-inflammatory DP (*n* = 2); study assessed the association between olive (EVOO vs washed olive oil) on background of a Mediterranean DP and inflammatory and therefore did not meet the eligibility criteria for inclusion in the current review (*n* =1). |
| Neale 2016^25^ |  | 17 | 15 | Primary studies did not meet the eligibility criteria: (i) meta-analysis combined multiple DPs including DASH, Tibetan, therefore effect of specific DP could not be separated out to specific DPs (*n* = 2). |
| Nomikos 2018^26^ | SR | 17 | Review excluded | Review excluded due to 100% overlap of eligible primary studies (*n* = 3). All other primary studies did not meet the eligibility criteria: (i) assessed association between specific food components and inflammatory biomarkers outcomes (*n* = 11); (ii) assessed association between alcohol and inflammatory biomarkers (*n* = 3). |
| Nordmann 201^27^ | SRMA | 7 | 5 | Review authors conducted a meta-analysis that included 5 eligible primary studies. All other primary studies were not included in the review synthesis assessing the effect of DP on markers of inflammation (*n* = 2). |
| Ojo 2019^28^ |  | 9 | 5 | Primary studies did not meet the eligibility criteria: (i) did not assess the association between dietary interventions and measured outcomes inflammatory markers (*n* = 4) |
| Philippou 2021^29^ | SR | 70 | 5 | Primary studies did not meet the eligibility criteria: (i) did not assess the association between dietary interventions and measured outcomes inflammatory markers (n = 36); (ii) assessed the association between single foods, food components or supplements and inflammatory biomarkers (n = 18); (iii) assessed the association between elimination/ allergy restricted diets and inflammatory biomarkers outcomes (n = 5); (iii) assessed the association between elemental diets and inflammatory markers (*n* = 3); (iv) study duration <4 weeks (*n* = 2); (v) intervention included 3 diet phases including fasting and effects on inflammatory markers can not be separated out due to lack of control (*n* = 1) |
| Pickworth 2019^30^ | SR | 56 | 29 | Review authors provided a narrative synthesis that included 35 primary studies (PS) deemed eligible for inclusion in for the current umbrella review. All other PS did not meet the eligibility criteria: (i) assessed the association between specific foods/ food components and inflammatory biomarkers outcomes (*n* = 17); (ii) included in supplemental table 1 but not clearly summarised/ detailed in the narrative synthesis (*n* = 6); (iii) Eligible studies had 100% overlap with another review (n = 4) |
| Pourrajab 2024^31^ | SRMA | 15 | 14 | Review authors conducted narrative and meta-analysis that included the findings of 15 eligible primary studies. The authors did not report the outcome findings for a single and therefore this PS was not included in the current umbrella review. |
| Rocha 2016^32^ | SR | 9 | Review excluded | Primary studies did not meet the eligibility criteria: (i) 100 % overlap with another review (*n* = 1); (ii) assessed association between food components and inflammatory biomarkers outcomes (*n* = 7); (iii) assessed the association between foods/ food groups and inflammatory biomarkers (*n* = 1). |
| Rondanelli 2024 | SRMA | 7 | 5 | Review authors conducted meta-analyses that included 5 eligible RCTs. The remaining studies were not included in the narrative synthesis nor any meta-anlyses (n = 2) |
| Sanchez-Rosales 2022^33^ |  | 10 | 4 | Review authors conducted narrative and/ or meta-analysis that included 4 eligible primary studies. All other primary studies did not meet the eligibility criteria: (i) intervention included replacement meal therapy (*n* = 1); (ii) assessed effect of single foods, nutrients or energy density on markers of inflammation (*n* = 2); (iii) did not assess the association between dietary interventions and measured outcomes inflammatory markers (*n* = 3). |
| Schönenberger 2021^34^ |  | 12 | Review excluded | The meta-analysis combined several different DPs including Mediterranean, vegetarian, anti-inflammatory and gluten free DPs, therefore effect of specific DP could not be separated out. The review does not meet the inclusion criteria of the current umbrella review which seeks to determine the evidence for specific dietary patterns. |
| Schwingshackl 2013^35^ |  | 14 | 5 | Primary studies were not reported in the review synthesis or meta-analysis (*n = 9*). |
| Soltani 2018^36^ | SRMA | 7 | 4 | The duration of a single primary study did not meet the ≥4 week inclusion criteria, therefore all results from all meta-analyses that included this study were not reported in the current umbrella review (*n* = 3) |
| Steckhan 2016^37^ | SRMA | 13 | 3 | Primary studies were not reported in the review synthesis or meta-analysis (*n =* 8). Meta-analyses conducted for CRP, TNF-α and IL-6 include primary studies that did not meet the eligibility criteria: assessed effects of single food groups (*n* = 2) |
| Vilela 2021^38^ | SR | 7 | Review excluded | Eligible primary studies (*n* = 2) were not included in the qualitative (narrative) synthesis reporting the effects of dietary patterns on markers of inflammation. All other primary studies did not report inflammatory biomarkers outcomes (*n =* 5) |
| Wu 2021^39^ | SRMA | 13 | 8 | All other primary studies were not included in the review synthesis/ meta-analysis assessing the effect of DP on markers of inflammation (*n* = 5). |
| Yeh 2021^40^ | SR | 17 | 5 | Primary studies did not meet the eligibility criteria: (i) 100% overlap for single eligible primary study that assessed the effect of DASH DP on markers of inflammation (*n* = 1); (ii) utilised dietary scores/ indices that assessed dietary intake of nutrients and single foods (*n* = 10); (iii) assessed the effects of dietary advice as the intervention (*n* = 1). |

**Supplemental Table S4.** Summary of methods used to manage dietary patterns in included reviews

| **Review reference and type** | **Methods to assess dietary intake** | **Methods of determining dietary patterns** |
| --- | --- | --- |
| Aleksandrova 2021^3^  Systematic review | Observational studies: Food frequency questionnaire (n = 2), diet record (n = 1), 24-h diet recall (n = 1)  Intervention trials: dietary intake questionnaire or diet record to evaluate compliance with intervention diet | *Definition:* Review authors did not provide an overall definition of the included dietary patterns in methods/results.  *Dietary pattern construction:* A priori approach (diet quality scores/ indices) (n = 3). Methods used to construct/ assess dietary patterns, in primary studies, were not reported in the methods/results of the included review. |
| Bujtor 2021^5^  Systematic review | Not reported | *Definition:* Review authors did not provide an overall definition of the included dietary patterns in methods/results  *Dietary pattern construction:* Methods used to construct/ assess dietary patterns, in primary studies, were not reported in the methods/results of the included review. |
| Chiavaroli 2018^41^  Systematic review and meta-analysis | Not reported | *Definition:* Definition of Portfolio dietary pattern provided.  *Dietary pattern construction:* Methods used to construct/ assess dietary patterns, in primary studies, were not reported in the methods/results of the included review. |
| Chiavaroli 2021^6^  Systematic review and meta-analysis | Food frequency questionnaire (n = 1), 3-d food record (n = 2), 24-h diet recall (n = 1), not reported (n = 2). Number and frequency of dietary intake assessments varied widely but were reported for 3of 6 included primary studies | *Definition:* Review authors did not provide an overall definition of the included dietary patterns in methods/results  *Dietary pattern construction:* Methods used to construct/ assess dietary patterns, in primary studies, were not reported in the methods/results of the included review. |
| Craddock 2019^8^  Systematic review and meta-analysis | Not reported | *Definition:* Review authors did not provide an overall definition of the included dietary patterns in methods/results  *Dietary pattern construction:* Methods used to construct/ assess dietary patterns, in primary studies, were not reported in the methods/results of the included review. |
| English 2022^12^  Systematic review | Not reported | *Definition:* Review authors did not provide an overall definition of the included dietary patterns in methods/results  *Dietary pattern construction:* Methods used to construct/ assess dietary patterns, in primary studies, were not reported in the methods/results of the included review. |
| Grammatikopoulou 2020^14^  Systematic review | Food frequency questionnaire (n = 1), food diary (n = 2) | *Definition:* Review authors did not provide an overall definition of the included dietary patterns in methods/results  *Dietary pattern construction:* Methods used to construct/ assess dietary patterns, in primary studies, were not reported in the methods/results of the included review. |
| Haghighatdoost 2017^15^  Systematic review and meta-analysis | Not reported | *Definition:* Review authors did not provide an overall definition of the included dietary patterns in methods/results  *Dietary pattern construction:* Methods used to construct/ assess dietary patterns, in primary studies, were not reported in the methods/results of the included review. |
| Ji 2025^42^  Systematic review and meta-analysis | Not reported | *Definition:* Review authors defined the Ketogenic diet within the eligibility criteria and the intervention diet is well described in the results table. The types of foods consumed as part of the ketogenic diet were not described methods/results  *Dietary pattern construction:* macro-nutrient ratio used to devise dietary pattern |
| Koelman 2022^20^  Systematic review and meta-analysis | Not reported | *Definition:* Review authors did not provide an overall definition of the included dietary patterns in methods/results  *Dietary pattern construction:* Methods used to construct/ assess dietary patterns, in primary studies, were not reported in the methods/results of the included review. |
| Massara 2022^22^  Systematic review and meta-analysis | Not reported | *Definition:* Review authors did not provide an overall definition of the included dietary patterns in methods/results  *Dietary pattern construction:* Methods used to construct/ assess dietary patterns, in primary studies, were not reported in the methods/results of the included review. |
| Mayr 2018^43^  Systematic review and meta-analysis | Food frequency questionnaire, Mediterranean diet scores | *Definition:* Review authors did not provide an overall definition of the included dietary patterns in methods/results  *Dietary pattern construction:* Review authors described a single cross-sectional study that used principal components analysis to determine a semi-Mediterranean diet pattern. |
| Menzel 2020^44^  Systematic review and meta-analysis | Not reported | *Definition:* Review authors did not provide an overall definition of the included dietary patterns in methods/results  *Dietary pattern construction:* Methods used to construct/ assess dietary patterns, in primary studies, were not reported in the methods/results of the included review. |
| Moore 2022^45^  Systematic review | Food frequency questionnaire (n = 2), dietary adherence score (n = 3), dietary intake questionnaire (n = 1), diet record (n = 1) | *Definition:* Review authors did not provide an overall definition of the included dietary patterns in methods/results  *Dietary pattern construction:* Methods used to construct/ assess dietary patterns, in primary studies, were not reported in the methods/results of the included review. |
| Mukherjee 2022^24^  Systematic review | Food frequency questionnaire (n = 3), dietary adherence score (Mediterranean diet score) (n = 3), 3-d food record (n = 2), 3-d weighted food record (n = 1), 7-d food record (n= 3),24-h diet recall (n = 1), nutritional survey (n = 2) | *Definition:* Review authors did not provide an overall definition of the included dietary patterns in methods/results  *Dietary pattern construction:* Methods used to construct/ assess dietary patterns, in primary studies, were not reported in the methods/results of the included review. |
| Neale 2016^25^  Systematic review and meta-analysis | Not reported | *Definition:* Review authors did not provide an overall definition of the included dietary patterns in methods/results  *Dietary pattern construction:* Methods used to construct/ assess dietary patterns, in primary studies, were not reported in the methods/results of the included review. |
| Nordmann 2011^46^  Systematic review and meta-analysis | Not reported | *Definition:* Review authors did not provide an overall definition of the included dietary patterns in methods/results  *Dietary pattern construction:* Methods used to construct/ assess dietary patterns, in primary studies, were not reported in the methods/results of the included review. |
| Ojo 2019^28^  Systematic review and meta-analysis | Not reported | *Definition:* Review authors did not provide an overall definition of the included dietary patterns in methods/results  *Dietary pattern construction:* Methods used to construct/ assess dietary patterns, in primary studies, were not reported in the methods/results of the included review. |
| Philippou 2021^29^  Systematic review | Food diary, 24-h diet recall | *Definition:* Review authors did not provide an overall definition of the included dietary patterns in methods/results  *Dietary pattern construction:* Methods used to construct/ assess dietary patterns, in primary studies, were not reported in the methods/results of the included review. |
| Pickworth 2019^30^  Systematic review | Not reported | *Definition:* Review authors did not provide an overall definition of the included dietary patterns in methods/ results, except for the Mediterranean and DASH diets.  *Dietary pattern construction:* Methods used to construct/ assess dietary patterns, in primary studies, were not reported in the methods/results of the included review. |
| Pourrajab 2024^31^ | 24-h diet recall (inconsistently reported) | *Definition:* Review authors provided a definition of the Mediterranean dietary patterns in the results (general guidelines recommendations for included PS).  *Dietary pattern construction:* Methods used to construct/ assess dietary patterns, in primary studies, were not reported in the methods/results of the included review |
| Rondanelli 2024^47^  Systematic review and meta-analysis | Not reported | *Definition:* Review authors did not provide an overall definition of the included dietary patterns in methods/results  *Dietary pattern construction:* Methods used to construct/ assess dietary patterns, in primary studies, were not reported in the methods/results of the included review. |
| Sakhaei 2019^48^  Systematic review and meta-analysis | Not reported | *Definition:* Review authors reported a definition of the Nordic dietary patterns in the results.  *Dietary pattern construction:* Methods used to construct/ assess dietary patterns, in primary studies, were not reported in the methods/results of the included review. |
| Sánchez-Rosales 2022^33^  Systematic review and meta-analysis | Food diaries (n = 1), Food frequency questionnaire (n = 4) dietary score (n = 1), 3-d food diary (n = 1), 7-d food record (n = 1), dietary intake questionnaire (n = 1), | *Definition:* Review authors did not provide an overall definition of the included dietary patterns in methods/results  *Dietary pattern construction:* Methods used to construct/ assess dietary patterns, in primary studies, were not reported in the methods/results of the included review. |
| Schwingshackl 2013^35^  Systematic review and meta-analysis | Not reported | *Definition:* Review authors did not provide an overall definition of the included dietary patterns in methods/results  *Dietary pattern construction:* Methods used to construct/ assess dietary patterns, in primary studies, were not reported in the methods/results of the included review. |
| Schwingshackl 2014^49^  Systematic review and meta-analysis | Not reported | *Definition:* Review authors did not provide an overall definition of the included dietary patterns in methods/results  *Dietary pattern construction:* Methods used to construct/ assess dietary patterns, in primary studies, were not reported in the methods/results of the included review. |
| Steckhan 2016^37^  Systematic review and meta-analysis | Not reported | *Definition:* Review authors did not provide an overall definition of the included dietary patterns in methods/results  *Dietary pattern construction:* Methods used to construct/ assess dietary patterns, in primary studies, were not reported in the methods/results of the included review. |
| Wu 2021^39^  Systematic review and meta-analysis | Food frequency questionnaire (n = 7) | *Definition:* Review authors reported brief definition of the Mediterranean dietary patterns included primary studies  *Dietary pattern construction:* Methods used to construct/ assess dietary patterns, in primary studies, were not reported in the methods/results of the included review. |
| Yeh 2021^40^  Systematic review | Dietary intake questionnaire (n = 1), Food frequency questionnaire (n = 2), 24-h diet recall (n = 1), 3-d food record (n = 1). Authors provide details of number and frequency of dietary assessments conducted in the relevant primary studies with wide variability between the studies. | *Definition:* Review authors did not provide an overall definition of the included dietary patterns in methods/results  *Dietary pattern construction:* Methods used to construct/ assess dietary patterns, in primary studies, were not reported in the methods/results of the included review. |

|  | |  | | | |  | |  | | |  |  |  | |  |  |
| --- | --- | --- | --- | --- | --- | --- | --- | --- | --- | --- | --- | --- | --- | --- | --- | --- |
| **Supplemental Table S5.**  Characteristics of the individual eligible meta-analyses and the certainty of evidence for the relationships between dietary patterns (relative to the comparator diet) and inflammatory biomarkers. | | | | | | | | | | | | | | | | |
| **Review** | | **Design** | | | | **Sample Size (n)** | | **Health Status** | | | **Comparator Diets*** | **MD (95% CI)** | **SMD (95% CI)**  **Effect Estimate** | | **I^2^** | **CoE** |
|  | | | | | | | | |  |  |  |  |  |  |  |  |
| **C-reactive protein (mg/L)** | | | | | | | | |  |  |  |  |  |  |  |  |
| MEDITERRANEAN DIETARY PATTERN | | | | | | | | | | | |  |  | |  |  |
| Koelman 2022 | RCT | | | | 11 (1805) | | Unspecified | | | LF; Habit | -1.00 [-2.02, 0.01] |  | | 97 | MODERATE |  |
| Mayr 2017 | IT | | | | 4 (1128) | | CHD | | | LF | -0.11 [-0.36, 0.15] |  | | 90 | MODERATE |  |
| Nordmann 2011 | RCT | | | | 5 (2435) | | OW;OB;CVD | | | LF | -0.97 [-1.49, -0.46] |  | | 82 | MODERATE |  |
| Neale 2016 | RCT | | | | 11 (1035) | | Healthy;OW;OB | | | LF;Habit;Healthy | -0.83 [-1.25, -0.40] |  | | 91 | LOW |  |
| Pourrajab 2023 | RCT | | | | 5 (739) | | CVD;HTN | | | LF |  | -0.87 (-1.49, -0.26) | | 89 | MODERATE |  |
| Pourrajab 2023 (hs-CRP) | RCT | | | | 5 (1537) | | CVD;NAFLD | | | LF |  | -1.04 (-1.80, -0.27) | | 97 | HIGH |  |
| Schwingshackl 2014 | IT | | | | 14 (1942) | | T2D;MetS;CVD | | | LF;Habit;Healthy | -0.98 [-1.48, -0.49] |  | | 91 | LOW |  |
| Sánchez-Rosales 2022 | RCT | | | | 3 (280) | | T2D | | | LF; Habit |  | -0.37 [-1.37, 0.64] | | 85 | MODERATE |  |
| Wu 2021 | CSS | | | | 6 (NR) | | CVD + elderly | | | Adherence |  | -0.26 [-0.41, -0.11] | | 79 | MODERATE |  |
| VEGETARIAN DIETARY PATTERN | | | | | | | | | | | |  |  | |  |  |
| Koelman 2022 | RCT | | | | 4 (413) | | Unspecified | | | Habit;AHA | -0.78 [-1.80, 0.25] |  | | 97 | MODERATE |  |
| Menzel 2020 | CSS | | | | 14 (7099) | | Healthy | | | Omni | -0.25 [-0.49, 0.00] |  | | 80 | LOW |  |
| Menzel 2020 | CSS | | | | 4 (674) | | IKF | | | Omni | -3.91 [-5.23, -2.60] |  | | 58 | LOW |  |
| Craddock 2019 | CSS | | | | 18 (6580) | | Unspecified | | | Omni | -0.61 [-0.91, -0.32] |  | | 100 | VERY LOW |  |
| Haghighatdoost 2017 | CSS | | | | 17 (NR) | | Unspecified | | | Omni |  | -0.15 [-0.35, 0.05] | | 76 | VERY LOW |  |
| Haghighatdoost 2017 (≥6 m) | CSS | | | | 6 (NR) | | Unspecified | | | Omni |  | 0.00 [-0.17, 0.17] | | 48 | VERY LOW |  |
| Haghighatdoost 2017 (≥2 y) | CSS | | | | 9 (NR) | | Unspecified | | | Omni |  | -0.29 [-0.59, 0.01] | | 69 | VERY LOW |  |
| NORDIC DIETARY PATTERN | | | | | | | | |  | | |  |  | |  |  |
| Massara 2022 | RCT | | | | 5 (606) | | OB;OW;MetS | | | Habit; Healthy | -1.91 [-6.37, 2.55] |  | | 66 | MODERATE |  |
| Sakhaei 2019 | RCT | | | | 7 (613) | | OB;MetS | | | Habit;Paleo | -0.17 [-0.69, 0.35] |  | | 68 | MODERATE |  |
| Neale 2016 | RCT | | | | 2 (300) | | Healthy;OW;OB | | | Habit | -1.18 [-4.36, 2.00] |  | | 93 | LOW |  |
| LOW GLYCAEMIC DIETARY PATTERN | | | | | | | | |  | | |  |  | |  |  |
| Chiavaroli 2021 | RCT | | | | 6 (622) | | T2D | | | HG;LF;LFibre | -0.41 [-0.78, -0.04] |  | | 24 | MODERATE |  |
| Ojo 2019^§^ | RCT | | | | 5 (420) | | T2D | | | HG | -0.32 [-1.17, 0.53] |  | | 0 | MODERATE |  |
| Schwingshackl 2013 | RCT | | | | 7 (1204) | | OB;OW | | | HG;LF;LFibre | -0.43 [-0.78, -0.09] |  | | 0 | LOW |  |
| DASH DIETARY PATTERN | | | | | | | | |  | | |  |  | |  |  |
| Koelman 2022 | RCT | | | | 4 (348) | | Unspecified | | | Habit | -0.27 [-0.62, 0.08] |  | | 72 | MODERATE |  |
| Soltani 2018 (≥8 w) | RCT | | | | 4 (380) | | T2D; NAFLD | | | Habit;Portfolio | -0.74 [-1.36, -0.11] |  | | 93 | MODERATE |  |
| VEGAN DIETARY PATTERN | | | | | | |  | |  | | |  |  | |  |  |
| Menzel 2020 | CSS | | | | 3 (266) | | Healthy | | | Omni | -0.54 [-0.79, -0.28] |  | | 0 | LOW |  |
| Craddock 2019 | CSS | | | | 5 (317) | | Unspecified | | | Omni | -0.13 [-0.29, 0.02] |  | | 87 | VERY LOW |  |
| PORTFOLIO DIETARY PATTERN | | | | | | |  | |  | | |  |  | |  |  |
| Chiavaroli 2018 | IT | | | | 7 (435) | | HLD | | | NCEP Step II | -0.58 [-1.01, -0.15] |  | | 33 | MODERATE |  |
| KETOGENIC DIETARY PATTERN | |  | | | |  | |  | | |  |  |  | |  |  |
| Ji 2025 | RCT | | | | 15 (909) | | OW;OB;T2D | | | WFD;LF;Habit | -0.16 [-1.31, 0.98] |  | | 96 | MODERATE |  |
| Rondanelli 2024 | RCT | | | | 4 (151) | | OW;OB;PD | | | WFD;MED | -0.62 [-0.84, -0.40] |  | | 43 | LOW |  |
| LOW CARB DIETARY PATTERN | | |  | | | |  | |  | | |  |  | |  |  |
| Steckhan 2017 | RCT | | | | 3 (126) | | MetS | | | LF; Habit |  | 0.44 [-0.47, 1.34] | | 76 | LOW |  |
| LOW FAT DIETARY PATTERN | | |  | | | |  | |  | | |  |  | | | |
| Steckhan 2016 | RCT | | | | 2 (48) | | MetS | | | LC |  | -0.98 [–1.60, -0.35] | | 0 | LOW |  |
|  | |  | | | |  | |  | | |  |  |  | |  |  |
| **Interleukin-6 (pg/mL)** | | |  | | | |  | |  | | |  |  | | | |
| MEDITERRANEAN DIETARY PATTERN | | | | | | |  | |  | | |  |  |  |  |  |
| Koelman 2022 | RCT | | | | 8 (524) | | Unspecified | | | LF;Habit | -1.07 [-1.94, -0.20] |  | | 96 | MODERATE |  |
| Pourrajab 2023 | RCT | | | | 13 (2663) | | CVD;NAFLD | | | LF |  | -0.38 (-0.55, -0.21) | | 71 | LOW |  |
| Schwingshackl 2014 | IT | | | | 6 (1077) | | T2D;MetS;CVD | | | LF;Habit;Healthy | -0.42 [-0.73, -0.11] |  | | 81 | LOW |  |
| LOW GLYCAEMIC DIETARY PATTERN | | | | | |  |  | |  | | |  |  | | | |
| Ojo 2019^§^ | RCT | | | | 2 (191) | | T2D | | | HG | -1.01 [-1.55, -0.48] |  | | 0 | MODERATE |  |
| NORDIC DIETARY PATTERN | | |  | | | |  | |  | | |  |  | | | |
| Sakhaei 2019 | RCT | | | | 3 (205) | | OB;MetS | | | Habit;Paleo | 0.13 [-0.29, 0.56] |  | | NR | MODERATE |  |
| VEGETARIAN DIETARY PATTERN | | |  | | | |  | |  | | |  |  | | | |
| Haghighatdoost 2017 | CSS | | | | 3 (401) | | Unspecified | | | Omni |  | 0.21 [0.18, 0.25] | | 0 | VERY LOW |  |
| KETOGENIC DIETARY PATTERN | |  | | | |  | |  | | |  |  |  | |  |  |
| Ji 2025 | RCT | | | | 7 (239) | | OW;OB;T2D | | | WFD;LF;Habit | -0.19 [-0.56, 0.18] |  | | 20 | MODERATE |  |
| Rondanelli 2024 | RCT | | | | 2 (45) | | OW;OB;PA | | | MED | -1.31 [-2.86, 0.25] |  | | 0 | VERY LOW |  |
|  | |  | | | |  | |  | | |  |  |  | |  |  |
| **TNF-α (pg/mL)** | | |  | | | |  | |  | | |  |  | | | |
| MEDITERRANEAN DIETARY PATTERN | | | | | | |  | |  | | |  |  |  |  |  |
| Koelman 2022 | RCT | | | | 4 (335) | | Unspecified | | | LF;Habit | -1.69 [-3.40, 0.02] |  | | 89 | MODERATE |  |
| Neale 2016 | RCT | | | | 3 (234) | | Healthy;OW;OB | | | Habit | 0.17 [-0.38, 0.72] |  | | 0 | MODERATE |  |
| Pourrajab 2023 | RCT | | | | 7 (1242) | | CVD;NAFLD | | | LF |  | -0.28 (-0.51, -0.05) | | 62 | VERY LOW |  |
| NORDIC DIETARY PATTERN | | |  | | | |  | |  | | |  |  | | | |
| Sakhaei 2019 | RCT | | | | 3 (195) | | OB;MetS | | | Habit;Paleo | 0.23 [-0.75, 1.21] |  | | NR | MODERATE |  |
| KETOGENIC DIETARY PATTERN | |  | | | |  | |  | | |  |  |  | |  |  |
| Ji 2025 | RCT | | | | 4 (154) | | OW;OB;T2D | | | WFD;LF;Habit | -0.23 [-1.12, 0.66] |  | | 30 | MODERATE |  |
|  | |  | | | |  | |  | | |  |  |  | |  |  |
| **E-selectin (ng/mL)** | | |  | | | |  | |  | | |  |  | | | |
| MEDITERRANEAN DIETARY PATTERN | | | | |  | |  | |  | | |  |  |  |  |  |
| Koelman 2022 | RCT | | | | 3 (206) | | Unspecified | | | LF;Habit | -2.20 (-7.43, 3.02) |  | | 93 | MODERATE |  |
| Pourrajab 2023 | RCT | | | | 4 (223) | | CVD;NAFLD | | | LF |  | -0.09 (-0.55, 0.36) | | 62 | LOW |  |
| Schwingshackl 2014 | IT | | | | 2 (161) | | T2D;MetS;CVD | | | LF;Habit;Healthy | -0.67 (-6.51, 5.16) |  | | 20 | LOW |  |
|  |  | | | |  | |  | | |  |  |  | |  |  |  |
| **IL-8 (pg/mL)** | |  | | | |  | |  | | |  |  |  | |  |  |
| MEDITERRANEAN DIETARY PATTERN | | | | | | | |  | | |  |  |  | |  |  |
| Koelman 2022 | RCT | | | | 5 (229) | | Unspecified | | | LF;Habit | -1.34 (-2.96, 0.14) |  | | 88 | MODERATE |  |
| KETOGENIC DIETARY PATTERN | |  | | | |  | |  | | |  |  |  | |  |  |
| Ji 2025 | RCT | | | | 6 (177) | | OW;OB;T2D | | | WFD;LF;Habit | -0.11 [-2.85, 2.64] |  | | NR | MODERATE |  |
|  | |  | | | |  | |  | | |  |  |  | |  |  |
| **Other pro-inflammatory biomarkers** | | | |  | |  | |  | | |  |  |  | |  |  |
| MEDITERRANEAN DIETARY PATTERN | | | | | |  |  | |  | | |  |  | | | |
| **Il-1ß (pg/mL)** | | |  | | | |  | |  | | |  |  | | | |
| Koelman 2022 | RCT | | | | 3 (178) | | Unspecified | | | LF;Habit | -0.49 (-0.66, -0.25) |  | | 0 | MODERATE |  |
| **ICAM-1 (ng/mL)** | | |  | | | |  | |  | | |  |  | | | |
| Pourrajab 2023 | RCT | | | | 8 (2342) | | CVD;NAFLD | | | LF |  | -0.69 (-0.96, -0.43) | | 87 | MODERATE |  |
| Schwingshackl 2014 | IT | | | | 2 (586) | | Unspecified | | | LF;Habit | -23.73 (-41.24, -6.22) |  | | 34 | LOW |  |
| **P-selectin (ng/mL)** | |  | | | |  | |  | | |  |  |  | |  |  |
| Pourrajab 2023 | RCT | | | | 5 (267) | | CVD;NAFLD | | | LF |  | -0.78 (-1.06, -0.51) | | 16 | HIGH |  |
| **IFN-γ (pg;mL)** | | |  | | | |  | |  | | |  |  | | | |
| Koelman 2022 | RCT | | | | 3 (178) | | Unspecified | | | LF;Habit | -1.19 (-2.91, 0.53) |  | | 98 | MODERATE |  |
| **VCAM-1 (ng/mL)** | |  | | | |  | |  | | |  |  |  | |  |  |
| Pourrajab 2023 | RCT | | | | 7 (2067) | | CVD;NAFLD | | | LF |  | -0.29 (-0.46, -0.11) | | 62 | LOW |  |
|  | | |  | | | |  | |  | | |  |  | | | |
| VEGETARIAN DIETARY PATTERN | | |  | | | |  | |  | | |  |  | | | |
| **Fibrinogen (g/L)** | | |  | | | |  | |  | | |  |  |  |  |  |
| Craddock 2019 | CSS | | | | 3 (208) | | Unspecified | | | Omni | -0.22 (-0.41, -0.04) |  | | 17 | VERY LOW |  |
|  | |  | | | |  | |  | | |  |  |  | |  |  |
| **Adiponectin (anti-inflammatory) (μg/mL)** | | | | | | | | |  | | |  |  | | | |
| MEDITERRANEAN DIETARY PATTERN | | | | | | | | |  | | |  |  |  |  |  |
| Neale 2016 | RCT | | | | 4 (353) | | Healthy;OW;OB | | | LF;Habit;Healthy | 0.59 [-0.82, 2.00] |  | | 25 | MODERATE |  |
| Schwingshackl 2014 | IT | | | | 2 (286) | | MetS | | | LF;Healthy | 1.69 [ 0.27, 3.11] |  | | 78 | LOW |  |
| Sanchez-Rosales 2022 | RCT | | | | 3 (367) | | MetS | | | LF;Healthy |  | 0.88 (0.14, 1.62) | | 81 | LOW |  |
| LOW GLYCAEMIC DIETARY PATTERN | | | | | |  |  | |  | | |  |  | | | |
| Ojo 2019^§^ | RCT | | | | 5 (420) | | T2D | | | HG | 0.01 [ 0.00, 0.03] |  | | 0 | MODERATE |  |
|  | |  | | | |  | |  | | |  |  |  | |  |  |
| Abbreviations: CI, confidence interval; CoE, certainty of evidence; CSS, cross sectional study; CHD, coronary heart disease; CVD, cardiovascular disease; GDM, gestational diabetes mellitus; Habit, habitual; HG, high glycaemic; HLD, hyperlipidaemia; I^2^, heterogeneity; IKF, impaired kidney function; IT, intervention trial; LF, low-fat; LFibre, low fibre; MD, mean difference; MED, Mediterranean Diet; MetS, metabolic syndrome; OB, obesity; Omni; omnivorous; OW, overweight; Paleo, paleolithic; PA, psoriatic arthritis; PD, Parkinson's disease; RCT, randomised controlled trial; T2D, type 2 diabetes mellitus; WFD, whole food diet.  * the listed comparator diets encompass all diets evaluated against the intervention dietary pattern, across all primary studies included in the meta-  analysis.  ^§^ Indicates meta-analyses that applied fixed-effects model | | | | | | | | | | | | | | | | |
|  | | | | | | | | | | | | | | | | |

## Supplemental Figures

| Domain | 1 | **2** | 3 | **4** | 5 | 6 | **7** | 8 | **9** | 10 | **11** | 12 | **13** | 14 | **15** | 16 | Overall  quality |
| --- | --- | --- | --- | --- | --- | --- | --- | --- | --- | --- | --- | --- | --- | --- | --- | --- | --- |
| Aleksandrova 2021 | Y | PY | Y | PY | Y | Y | Y | Y | Y | N | N/A | N/A | Y | Y | N/A | Y | HIGH |
| Bujtor 2021 | Y | PY | Y | PY | N | N | Y | Y | Y | N | N/A | N/A | Y | Y | N/A | Y | MOD |
| Chiavaroli 2018 | Y | Y | Y | PY | Y | Y | Y | PY | Y | Y | Y | N | Y | Y | N/A | Y | HIGH |
| Chiavaroli 2021 | Y | Y | Y | PY | N | Y | Y | Y | Y | Y | Y | Y | Y | Y | N/A | Y | HIGH |
| Craddock 2019 | Y | PY | Y | PY | N | N | Y | PY | Y | N | Y | N | Y | Y | Y | Y | MOD |
| English 2022 | Y | Y | Y | PY | Y | Y | PY | Y | Y | N | N/A | N/A | Y | N | N/A | Y | MOD |
| Grammatiko. 2020 | Y | PY | Y | PY | Y | Y | Y | Y | Y | Y | N/A | N/A | Y | Y | N/A | Y | HIGH |
| Haghighatdo. 2017 | Y | N | N | PY | Y | N | Y | PY | Y | N | Y | N | N | Y | Y | Y | C/LOW |
| Ji 2025 | Y | PY | Y | Y | Y | N | Y | Y | Y | Y | Y | N | N | Y | Y | Y | LOW |
| Koelman 2022 | Y | PY | Y | PY | Y | Y | Y | PY | Y | N | Y | N | Y | Y | Y | Y | MOD |
| Massara 2022 | Y | Y | Y | PY | N | Y | Y | PY | Y | Y | Y | Y | Y | Y | Y | Y | HIGH |
| Mayr 2018 | Y | PY | Y | PY | N | N | Y | Y | Y | N | Y | N | Y | Y | N/A | Y | MOD |
| Menzel 2020 | Y | PY | Y | PY | Y | N | Y | PY | Y | N | Y | Y | Y | Y | Y | Y | MOD |
| Moore 2022 | Y | PY | Y | Y | Y | Y | Y | Y | Y | N | N/A | N/A | Y | Y | N/A | Y | MOD |
| Mukherjee 2022 | Y | PY | Y | Y | Y | Y | Y | Y | Y | N | N/A | N/A | Y | Y | N/A | Y | HIGH |
| Neale 2016 | Y | PY | Y | PY | N | N | Y | Y | Y | N | Y | Y | Y | Y | Y | Y | MOD |
| Nordmann 2011 | Y | N | Y | Y | Y | Y | Y | Y | PY | N | Y | Y | Y | Y | Y | Y | LOW |
| Ojo 2019 | Y | N | Y | PY | N | N | Y | PY | Y | N | Y | Y | Y | Y | N/A | Y | LOW |
| Philippou 2021 | Y | PY | Y | PY | Y | Y | PY | PY | Y | N | N/A | N/A | Y | Y | N/A | Y | LOW |
| Pickworth 2019 | Y | N | Y | PY | N | N | PY | PY | Y | N | N/A | N/A | Y | Y | N/A | Y | LOW |
| Pourrajab 2023 | Y | Y | Y | Y | Y | Y | Y | Y | Y | Y | Y | Y | Y | Y | Y | Y | HIGH |
| Rondanelli 2024 | Y | PY | Y | PY | Y | Y | PY | Y | Y | Y | Y | N | N | Y | Y | Y | LOW |
| Sakhaei 2019 | Y | PY | Y | PY | Y | Y | Y | Y | Y | N | Y | Y | Y | Y | Y | Y | HIGH |
| SánchezN/ARos. 2022 | Y | Y | Y | PY | Y | N | Y | PY | Y | N | Y | Y | Y | Y | Y | Y | MOD |
| Schwingshackl 2013 | Y | N | N | PY | N | N | Y | Y | Y | N | Y | N | N | Y | Y | Y | C/LOW |
| Schwingshackl 2014 | Y | N | Y | PY | N | N | Y | Y | Y | N | Y | Y | Y | Y | Y | Y | LOW |
| Soltani 2018 | Y | Y | Y | PY | Y | Y | Y | Y | Y | N | Y | Y | Y | Y | Y | Y | HIGH |
| Steckhan 2016 | Y | N | Y | PY | N | Y | Y | Y | Y | N | Y | Y | Y | Y | Y | Y | LOW |
| Wu 2021 | Y | Y | Y | PY | Y | Y | Y | Y | Y | N | Y | N | Y | Y | Y | Y | MOD |
| Yeh 2021 | Y | N | Y | PY | Y | Y | PY | PY | Y | N | N/A | N/A | N | Y | N/A | Y | C/LOW |
|  |  |  |  |  |  |  |  |  |  |  |  |  |  |  |  |  |  |
| Legend: Y, Yes; PY, Partial Yes; N, No; N/A, not applicable.  Grey shaded columns indicate critical domains (critical domains are Q2, Q4, Q7, Q9, Q11, Q13, Q15).  Domain definitions: Q1: Did the research questions and inclusion criteria for the review include the components of PICO?, Q2: 2. Did the report of the review contain an explicit statement that the review methods were established prior to the conduct of the review and did the report justify any significant deviations from the protocol?; Q3, Did the review authors explain their selection of the study designs for inclusion in the review?; Q4, Did the review authors use a comprehensive literature search strategy?; Q5, Did the review authors perform study selection in duplicate?; Q6, Did the review authors perform data extraction in duplicate?; Q7, Did the review authors provide a list of excluded studies and justify the exclusions?; Q8, Did the review authors describe the included studies in adequate detail?; Q9, Did the review authors use a satisfactory technique for assessing the risk of bias?; Q10, Did the review authors report on the sources of funding?; Q11, Did the review authors use appropriate methods for statistical combination of results?; Q12, Did the review authors assess the potential impact of RoB in individual studies on the results?; Q13, Did the review authors account for RoB in individual studies when interpreting/ discussing the results of the review?; Q14, Did the review authors provide a satisfactory explanation for, and discussion of, any heterogeneity?; Q15, Did the review authors carry out an adequate investigation of publication bias?; Q16, Did the review authors report any potential sources of conflict of interest. | | | | | | | | | | | | | | | | | |

### **Supplemental Figure S1.** Methodological quality of included reviews using AMSTAR 2 critical appraisal

|  |
| --- |

**Supplemental Figure S2.** AMSTAR methodological quality assessment of each item across the included reviews (critical domains are Q2, Q4, Q7, Q9, Q11, Q13, and Q15).

|  | Type of review | Primary study design | Risk of Bias | Inconsistency | Indirectness | Imprecision | Publication bias | Quality importance GRADE |
| --- | --- | --- | --- | --- | --- | --- | --- | --- |
| Chiavaroli 2018 | SRMA |  | □ | □ | □ | ■ | □ | Moderate |
| Chiavaroli 2021 | SRMA |  | □ | □ | □ | ■ | □ | Moderate |
| Massara 2022 | SRMA |  | □ | □ | □ | ■ | □ | Moderate |
| Neale 2016 (CRP) | SRMA |  | □ | ■ | □ | ■ | □ | Low |
| Neale 2016 (Other) | SRMA |  | □ | ■ | □ | ■ | □ | Moderate |
| Sánchez-Ros. 2022 (ApN) | SRMA |  | ■ | ■ | □ | □ | □ | Low |
| Sánchez-Ros. 2022 (CRP) | SRMA |  | ■ | ■ | □ | □ | □ | Moderate |

Downgrade is indicated by black shading. Total number of downgrades for each criterion is presumed to be 1 unless otherwise indicated by the superscript.

### **Supplemental Figure S3.** GRADE assessments as reported by authors of the included review

| Review | Type of review | Primary study design | Imprecision | Study quality | Inconsistency (I^2^) | Methodological quality | GRADE CoE |  |  |  |
| --- | --- | --- | --- | --- | --- | --- | --- | --- | --- | --- |
| Aleksandrova 2021 | SR | IT | □ | ■ | ■ | □ | MODERATE |  |  |  |
| Aleksandrova 2021 | SR | OS | □ | ■ | ■ | □ | VERY LOW^a^ |  |  |  |
| Bujtor 2021 | SR | OS | □ | □ | ■ | □ | LOW |  |  |  |
| Craddock 2019 | SRMA | IT | □ | ■ | ■ | □ | MODERATE |  |  |  |
| Craddock 2019 | SRMA | OS | □ | ■ | ■ | □ | VERY LOW^a^ |  |  |  |
| English 2022 | SRMA | IT | □ | □ | ■ | □ | MODERATE |  |  |  |
| English 2022 | SRMA | OS | □ | □ | ■ | □ | LOW^a^ |  |  |  |
| Grammatikopoulou 2020 | SRMA | IT | ■ | □ | ■ | □ | MODERATE |  |  |  |
| Haghighatdoost 2017 | SR | OS | □ | ■ | ■ | ■^2^ | VERY LOW^a^ |  |  |  |
| Ji 2025 | SRMA | IT | □ | □ | ■ | ■ | MODERATE |  |  |  |
| Koelman 2022 | SRMA | IT | □ | □ | ■ | □ | MODERATE |  |  |  |
| Mayr 2018 (CRP) | SR | IT | □ | □ | ■ | □ | MODERATE |  |  |  |
| Mayr 2018 (IL-6, TNF-α) | SR | IT | ■^2^ | □ | ■ | □ | LOW |  |  |  |
| Mayr 2018 (CRP, IL-6) | SR | OS | □ | □ | ■ | □ | VERY LOW^a^ |  |  |  |
| Mayr 2018 (TNF-α) | SR | OS | ■^2^ | □ | ■ | □ | VERY LOW^a^ |  |  |  |
| Menzel 2020 | SRMA | OS | □ | □ | ■ | □ | LOW |  |  |  |
| Menzel 2020 (ApN) | SRMA | OS | ■ | □ | ■ | □ | LOW |  |  |  |
| Moore 2022 | SRMA | IT | ■ | □ | ■ | □ | MODERATE |  |  |  |
| Mukherjee 2023 | SR | IT | □ | ■ | ■ | □ | MODERATE |  |  |  |
| Nordmann 2011 | SR | IT | □ | □ | ■ | ■ | MODERATE |  |  |  |
| Ojo 2019 | SRMA | IT | □ | □ | □ | ■ | MODERATE |  |  |  |
| Philippou 2021 (MED) | SRMA | IT | □ | ■ | ■ | ■ | LOW |  |  |  |
| Philippou 2021 (VGN) | SRMA | IT | ■^2^ | ■ | ■ | ■ | VERY LOW |  |  |  |
| Pickworth 2019 | SRMA | IT | □ | ■ | ■ | ■ | LOW |  |  |  |
| Pourrajab 2023 | SRMA | IT | □ | □ | ■ | □ | MODERATE |  |  |  |
| Rondanelli 2024 | SRMA | IT | ■ | □ | □ | ■ | MODERATE |  |  |  |
| Sakhaei 2019 | SRMA | IT | □ | □ | ■ | □ | MODERATE |  |  |  |
| Schwingshackl 2013 | SR | IT | □ | ■ | ■ | ■^2^ | LOW |  |  |  |
| Schwingshackl 2014 | SRMA | IT | □ | ■ | ■ | ■ | LOW |  |  |  |
| Soltani 2018 | SRMA | IT | □ | □ | ■ | □ | MODERATE |  |  |  |
| Steckhan 2016 | SRMA | IT | ■ | ■ | □ | ■ | LOW^b^ |  |  |  |
| Wu 2021 | SRMA | IT | □ | □ | ■ | □ | MODERATE |  |  |  |
| Wu 2021 | SRMA | OS | □ | □ | ■ | □ | LOW^a^ |  |  |  |
| Yeh 2021 | SR | OS | □ | □ | ■ | ■^2^ | VERY LOW^a^ |  |  |  |
| Downgrade is indicated by black shading. Total number of downgrades for each criterion is presumed to be 1 unless otherwise indicated by the superscript. | | | | | | | | | | |
| a | Observational studies default to a low GRADE of evidence due to the inherent risk of bias associated with the lack of randomization, namely confounding and selection bias, in accordance with Cochrane Handbook Chapter 14 ^50^. Further downgrades applied in accordance with the algorithm to determine overall certainty of evidence. | | | | | | | | |  |
| b | Sample sizes differed for inflammatory marker outcomes or dietary patterns, reported in review and therefore graded accordingly. | | | | | | | | |  |

### **Supplemental Figure S4.** Strength of evidence assessments using the GRADE algorithm.

|  | | **Review** | | **Health status** | | | **CRP** | | **IL-6** | | | **TNF-α** | | **Other** | | | **ApN** | |  |  |  |
| --- | --- | --- | --- | --- | --- | --- | --- | --- | --- | --- | --- | --- | --- | --- | --- | --- | --- | --- | --- | --- | --- |
| **Mediterranean dietary pattern** | | | |  | | |  | |  | | |  | |  | | |  | |  |  |  |
|  | *Intervention trials* | | |  | | |  | |  | | |  | |  | | |  | |  |  |  |
|  | | Aleksandrova 2021 | | H/OB/NAFLD | | | ▼^4^ | |  | | | ●^3^ | |  | | |  | |  |  |  |
|  | | English 2022 | | H/T2D/MetS | | |  | |  | | |  | | ●^4^ | | |  | |  |  |  |
|  | | Mayr 2018 | | CHD | | |  | | ● | | | ● | |  | | |  | |  |  |  |
|  | | Moore 2022 | | OB/OW | | | ●^2^ | | ●^4^ | | | ●^4^ | | ●^2^ | | |  | |  |  |  |
|  | | Mukherjee 2023 | | T2D/HTN/DLD | | | ▼^12^ | | ▼^8^ | | | ●^5^ | | ●^4^ | | |  | |  |  |  |
|  | | Philippou 2021 | | RA | | | ●^3^ | |  | | |  | |  | | |  | |  |  |  |
|  | | Pickworth 2019 | | OB/OW/T2D | | | ▼^4^ | |  | | |  | |  | | |  | |  |  |  |
|  | | Wu 2021 | | CAD+elderly | | | ▼^2^ | | ▼^2^ | | | ▼ | |  | | |  | |  |  |  |
|  | *Observational studies* | | |  | | |  | | | |  | | | |  | | | |  |  |  |
|  | | Aleksandrova 2021 | | H/OB/NAFLD | | | ▽^2^ | | ▽ | | | ▽ | | ▽ | | |  | |  |  |  |
|  | | Bujtor 2021 | | OB/OW | | | ●^9^ | |  | | |  | |  | | |  | |  |  |  |
|  | | Mayr 2018 | | CHD | | | ▽^3^ | | ▽ | | | ▽ | |  | | |  | |  |  |  |
|  | | Wu 2021 | | CAD+elderly | | | ▼ | | ● | | |  | |  | | |  | |  |  |  |
|  | | Yeh 2021 | | Pregnant | | | ▼ | |  | | |  | |  | | |  | |  |  |  |
| **Vegetarian dietary pattern** | | | |  | | |  | |  | | |  | |  | | |  | |  |  |  |
|  | *Intervention trials* | | |  | | |  | |  | | |  | |  | | |  | |  |  |  |
|  | | Craddock 2019 | | OB/OW/T2D | | | ▼ | | ● | | | ● | | ● | | |  | |  |  |  |
|  | *Observational studies* | | |  | | |  | |  | | |  | |  | | |  | |  |  |  |
|  | | Aleksandrova 2021 | | H/OB/NAFLD | | | ○ | |  | | |  | |  | | |  | |  |  |  |
|  | | Craddock 2019 | | CVD/T2D | | |  | | ○^3^ | | | ○^3^ | | ○^3^ | | |  | |  |  |  |
|  | | English 2022 | | H/T2D/MetS | | |  | |  | | |  | | ▼ | | |  | |  |  |  |
|  | | Menzel 2020 | | Healthy | | |  | | ▼^4^ | | | ● | | ● | | | ▼^2^ | |  |  |  |
| **Vegan dietary pattern** | | | |  | | |  | |  | | |  | |  | | |  | |  |  |  |
|  | *Intervention trials* | | |  | | |  | |  | | |  | |  | | |  | |  |  |  |
|  | | English 2022 | | H/T2D/MetS | | |  | |  | | |  | | ▼ | | |  | |  |  |  |
|  | | Philippou 2021 | | RA | | | ▽^8^ | |  | | |  | |  | | |  | |  |  |  |
|  | *Observational studies* | | |  | | |  | | | |  | | | |  | | | |  |  |  |
|  | | Menzel 2020 | | Healthy | | |  | |  | | |  | | ● | | | ● | |  |  |  |
| **Low glycaemic dietary pattern** | | | | | | |  | |  | | |  | |  | | |  | |  |  |  |
|  | *Intervention trials* | | |  | | |  | |  | | |  | |  | | |  | |  |  |  |
|  | | Bujtor 2021 | | OB/OW | | | ●^4^ | |  | | |  | |  | | |  | |  |  |  |
|  | | Pickworth 2019 | | OB/OW/T2D | | | ●^6^ | |  | | |  | |  | | |  | |  |  |  |
|  | | Yeh 2021 | | Pregnant | | | ○ | |  | | |  | |  | | |  | |  |  |  |
|  | *Observational studies* | | |  | | |  | | | |  | | | |  | | | |  |  |  |
|  | | Yeh 2021 | | Pregnant | | | ▽ | | ○ | | | ○ | |  | | |  | |  |  |  |
| **DASH dietary pattern** | | | |  | | |  | |  | | |  | |  | | |  | |  |  |  |
|  | *Observational studies* | | |  | | |  | |  | | |  | |  | | |  | |  |  |  |
|  | | Bujtor 2021 | | OB/OW | | | ●^2^ | |  | | |  | |  | | |  | |  |  |  |
| **HEI dietary pattern** | | | |  | | |  | | | |  | | | |  | | | |  |  |  |
|  | *Observational studies* | | |  | | |  | | | |  | | | |  | | | |  |  |  |
|  | | | Aleksandrova 2021 | | H/OB/NAFLD | | | ○ | |  | | |  | | |  | |  | |  |  |
|  | | Bujtor 2021 | | OB/OW | | | ●^3^ | |  | | |  | |  | | |  | |  |  |  |
| **High protein dietary pattern** | | | | | |  |  | |  | | |  | |  | | |  | |  |  |  |
|  | *Intervention trials* | | |  | | |  | |  | | |  | |  | | |  | |  |  |  |
|  | | Bujtor 2021 | | OB/OW | | | ● | |  | | |  | |  | | |  | |  |  |  |
|  | | Pickworth 2019 | | OB/OW/T2D | | | ●^4^ | |  | | |  | |  | | |  | |  |  |  |
| **Low carb. dietary pattern** | | | |  | | |  | |  | | |  | |  | | |  | |  |  |  |
|  | *Intervention trials* | | |  | | |  | |  | | |  | |  | | |  | |  |  |  |
|  | | Pickworth 2019 | | OB/OW/T2D | | | ●^14^ | |  | | |  | |  | | |  | |  |  |  |
| **Low FODMAP dietary pattern** | | | |  | | |  | |  | | |  | |  | | |  | |  |  |  |
|  | *Intervention trials* | | |  | | |  | |  | | |  | |  | | |  | |  |  |  |
|  | | Grammatik. 2020 | | IBD remission | | | ●^3^ | |  | | |  | |  | | |  | |  |  |  |
| **Paleo dietary pattern** | | | |  | | |  | |  | | |  | |  | | |  | |  |  |  |
|  | *Observational studies* | | |  | | |  | |  | | |  | |  | | |  | |  |  |  |
|  | | Aleksandrova 2021 | | H/OB/NAFLD | | | ▽ | |  | | |  | |  | | |  | |  |  |  |
| **Western dietary pattern** | | | |  | | |  | |  | | |  | |  | | |  | |  |  |  |
|  | *Observational studies* | | |  | | |  | |  | | |  | |  | | |  | |  |  |  |
|  | | Bujtor 2021 | | OB/OW | | | ▲^2^ | |  | | |  | |  | | |  | |  |  |  |
| Legend: Effect direction (overall effect on inflammatory biomarker outcomes): ▼ indicates a beneficial effect direction;  ● indicates a direction of no effect. The CoE for each synthesis is indicated by the tone of the symbol as follows: filled in black depicts ‘moderate’ CoE; filled in grey depicts ‘low’ CoE; filled in white depicts ‘very low’ CoE. Sample size (pooled participants in synthesis): very large symbol▼>1000; large arrow▼ 501-1000; medium arrow ▼ 100-500; small arrow ▼ <100. Superscript numbers: number of primary studies within each outcome synthesis is 1, unless indicated by superscript beside effect direction. | | | | | | | | | | | | | | | | | | | | | |

**Supplemental Figure S5.** Effect direction plot for dietary patterns on inflammatory marker outcomes

References

1. Abdallah J, Assaf S, Das A, Hirani V. Effects of anti-inflammatory dietary patterns on non-alcoholic fatty liver disease: a systematic literature review. *Eur J Nutr*. Jun 2023;62(4):1563-1578. doi:10.1007/s00394-023-03085-0

2. Akbar Z, Fituri S, Ouagueni A, et al. Associations of the MIND Diet with Cardiometabolic Diseases and Their Risk Factors: A Systematic Review. *Diabetes Metab Syndr Obes*. 2023;16:3353-3371. doi:10.2147/dmso.S427412

3. Aleksandrova K, Koelman L, Rodrigues CE. Dietary patterns and biomarkers of oxidative stress and inflammation: A systematic review of observational and intervention studies. *Redox Biol*. Jun 2021;42:101869. doi:10.1016/j.redox.2021.101869

4. Apekey TA, Maynard MJ, Kittana M, Kunutsor SK. Comparison of the Effectiveness of Low Carbohydrate Versus Low Fat Diets, in Type 2 Diabetes: Systematic Review and Meta-Analysis of Randomized Controlled Trials. *Nutrients*. Oct 19 2022;14(20)doi:10.3390/nu14204391

5. Bujtor M, Turner AI, Torres SJ, Esteban-Gonzalo L, Pariante CM, Borsini A. Associations of Dietary Intake on Biological Markers of Inflammation in Children and Adolescents: A Systematic Review. *Nutrients*. 2021;13(2):356.

6. Chiavaroli L, Lee D, Ahmed A, et al. Low Glycemic Index/Load Dietary Patterns and Glycemia and Cardiometabolic Risk Factors in Diabetes: A Systematic Review and Meta-Analysis of Randomized Controlled Trials. *Current Developments in Nutrition*. 2021/06/01/ 2021;5:1018. doi:<https://doi.org/10.1093/cdn/nzab053_011>

7. Cowan SF, Leeming ER, Sinclair A, Dordevic AL, Truby H, Gibson SJ. Effect of whole foods and dietary patterns on markers of subclinical inflammation in weight-stable overweight and obese adults: a systematic review. *Nutr Rev*. Jan 1 2020;78(1):19-38. doi:10.1093/nutrit/nuz030

8. Craddock JC, Neale EP, Peoples GE, Probst YC. Vegetarian-Based Dietary Patterns and their Relation with Inflammatory and Immune Biomarkers: A Systematic Review and Meta-Analysis. *Advances in Nutrition*. 2019;10(3):433-451. doi:10.1093/advances/nmy103

9. García Pérez de Sevilla G, Sánchez-Pinto Pinto B. Effectiveness of Workplace Mediterranean Diet Interventions on Cardiometabolic Risk Factors: A Systematic Review. *Workplace Health & Safety*. 2022/02/01 2022;70(2):73-80. doi:10.1177/21650799211045708

10. Dos Reis Padilha G, Sanches Machado d’Almeida K, Ronchi Spillere S, Corrêa Souza G. Dietary Patterns in Secondary Prevention of Heart Failure: A Systematic Review. *Nutrients*. 2018;10(7). doi:10.3390/nu10070828

11. Eichelmann F, Schwingshackl L, Fedirko V, Aleksandrova K. Effect of plant-based diets on obesity-related inflammatory profiles: a systematic review and meta-analysis of intervention trials. *Obes Rev*. Nov 2016;17(11):1067-1079. doi:10.1111/obr.12439

12. English CJ, Mayr HL, Lohning AE, Reidlinger DP. The association between dietary patterns and the novel inflammatory markers platelet-activating factor and lipoprotein-associated phospholipase A2: a systematic review. *Nutrition Reviews*. 2022;80(6):1371-1391. doi:10.1093/nutrit/nuab051

13. Ghaedi E, Mohammadi M, Mohammadi H, et al. Effects of a Paleolithic Diet on Cardiovascular Disease Risk Factors: A Systematic Review and Meta-Analysis of Randomized Controlled Trials. *Advances in nutrition (Bethesda, Md)*. 2019;10(4):634-646. doi:10.1093/advances/nmz007

14. Grammatikopoulou MG, Goulis DG, Gkiouras K, et al. Low FODMAP Diet for Functional Gastrointestinal Symptoms in Quiescent Inflammatory Bowel Disease: A Systematic Review of Randomized Controlled Trials. *Nutrients*. Nov 27 2020;12(12)doi:10.3390/nu12123648

15. Haghighatdoost F, Bellissimo N, Totosy de Zepetnek JO, Rouhani MH. Association of vegetarian diet with inflammatory biomarkers: a systematic review and meta-analysis of observational studies. *Public Health Nutr*. Oct 2017;20(15):2713-2721. doi:10.1017/s1368980017001768

16. Hart MJ, Torres SJ, McNaughton SA, Milte CM. Dietary patterns and associations with biomarkers of inflammation in adults: a systematic review of observational studies. *Nutrition Journal*. 2021;20(1)doi:10.1186/s12937-021-00674-9

17. Ilari S, Proietti S, Milani F, et al. Dietary Patterns, Oxidative Stress, and Early Inflammation: A Systematic Review and Meta-Analysis Comparing Mediterranean, Vegan, and Vegetarian Diets. *Nutrients*. Jan 31 2025;17(3)doi:10.3390/nu17030548

18. Kazeminasab F, Miraghajani M, Khalafi M, Sakhaei MH, Rosenkranz SK, Santos HO. Effects of low-carbohydrate diets, with and without caloric restriction, on inflammatory markers in adults: a systematic review and meta-analysis of randomized clinical trials. *Eur J Clin Nutr*. Jul 2024;78(7):569-584. doi:10.1038/s41430-024-01431-x

19. Klonizakis M, Bugg A, Hunt B, Theodoridis X, Bogdanos DP, Grammatikopoulou MG. Assessing the Physiological Effects of Traditional Regional Diets Targeting the Prevention of Cardiovascular Disease: A Systematic Review of Randomized Controlled Trials Implementing Mediterranean, New Nordic, Japanese, Atlantic, Persian and Mexican Dietary Interventions. *Nutrients*. 2021;13(9):3034.

20. Koelman L, Caue, Aleksandrova K. Effects of Dietary Patterns on Biomarkers of Inflammation and Immune Responses: A Systematic Review and Meta-Analysis of Randomized Controlled Trials. *Advances in Nutrition*. 2022;13(1):101-115. doi:10.1093/advances/nmab086

21. Lari A, Sohouli MH, Fatahi S, et al. The effects of the Dietary Approaches to Stop Hypertension (DASH) diet on metabolic risk factors in patients with chronic disease: A systematic review and meta-analysis of randomized controlled trials. *Nutr Metab Cardiovasc Dis*. Sep 22 2021;31(10):2766-2778. doi:10.1016/j.numecd.2021.05.030

22. Massara P, Zurbau A, Glenn AJ, et al. Nordic dietary patterns and cardiometabolic outcomes: a systematic review and meta-analysis of prospective cohort studies and randomised controlled trials. *Diabetologia*. Dec 2022;65(12):2011-2031. doi:10.1007/s00125-022-05760-z

23. Mayr HL, Tierney AC, Thomas CJ, Ruiz-Canela M, Radcliffe J, Itsiopoulos C. Mediterranean-type diets and inflammatory markers in patients with coronary heart disease: a systematic review and meta-analysis. *Nutr Res*. Feb 2018;50:10-24. doi:10.1016/j.nutres.2017.10.014

24. Mukherjee MS, Han CY, Sukumaran S, Delaney CL, Miller MD. Effect of anti-inflammatory diets on inflammation markers in adult human populations: a systematic review of randomized controlled trials. *Nutrition Reviews*. 2022:nuac045. PMID: 35831971. doi:10.1093/nutrit/nuac045

25. Neale EP, Batterham MJ, Tapsell LC. Consumption of a healthy dietary pattern results in significant reductions in C-reactive protein levels in adults: a meta-analysis. *Nutrition Research*. May 2016;36(5):391-401. doi:10.1016/j.nutres.2016.02.009

26. Nomikos T, Fragopoulou E, Antonopoulou S, Panagiotakos DB. Mediterranean diet and platelet-activating factor; a systematic review. *Clin Biochem*. Sep 2018;60:1-10. doi:10.1016/j.clinbiochem.2018.08.004

27. Nordmann AJ, Suter-Zimmermann K, Bucher HC, et al. Meta-Analysis Comparing Mediterranean to Low-Fat Diets for Modification of Cardiovascular Risk Factors. *The American Journal of Medicine*. 2011;124(9):841-851.e2. doi:10.1016/j.amjmed.2011.04.024

28. Ojo O, Ojo OO, Wang X-H, Adegboye ARA. The Effects of a Low GI Diet on Cardiometabolic and Inflammatory Parameters in Patients with Type 2 and Gestational Diabetes: A Systematic Review and Meta-Analysis of Randomised Controlled Trials. *Nutrients*. 2019;11(7):1584. doi:10.3390/nu11071584

29. Philippou E, Petersson SD, Rodomar C, Nikiphorou E. Rheumatoid arthritis and dietary interventions: systematic review of clinical trials. *Nutrition Reviews*. Apr 2021;79(4):410-428. doi:10.1093/nutrit/nuaa033

30. Pickworth CK, Deichert DA, Corroon J, Bradley RD. Randomized controlled trials investigating the relationship between dietary pattern and high-sensitivity C-reactive protein: a systematic review. *Nutr Rev*. Jun 1 2019;77(6):363-375. doi:10.1093/nutrit/nuz003

31. Pourrajab B, Fotros D, Asghari P, Shidfar F. Effect of the Mediterranean Diet Supplemented With Olive Oil Versus the Low-Fat Diet on Serum Inflammatory and Endothelial Indexes Among Adults: A Systematic Review and Meta-analysis of Clinical Controlled Trials. *NUTRITION REVIEWS*. 2024 NOV 12 2024;doi:10.1093/nutrit/nuae166

32. Rocha NP, Milagres LC, Longo GZ, Ribeiro AQ, Novaes JF. Association between dietary pattern and cardiometabolic risk in children and adolescents: a systematic review. *J Pediatr (Rio J)*. May-Jun 2017;93(3):214-222. doi:10.1016/j.jped.2017.01.002

33. Sánchez-Rosales AI, Guadarrama-López AL, Gaona-Valle LS, Martínez-Carrillo BE, Valdés-Ramos R. The Effect of Dietary Patterns on Inflammatory Biomarkers in Adults with Type 2 Diabetes Mellitus: A Systematic Review and Meta-Analysis of Randomized Controlled Trials. Review. *Nutrients*. 2022;14(21)4577. doi:10.3390/nu14214577

34. Schönenberger KA, Schüpfer A-C, Gloy VL, et al. Effect of Anti-Inflammatory Diets on Pain in Rheumatoid Arthritis: A Systematic Review and Meta-Analysis. *Nutrients*. 2021;13(12):4221. doi:10.3390/nu13124221

35. Schwingshackl L, Hoffmann G. Long-term effects of low glycemic index/load vs. high glycemic index/load diets on parameters of obesity and obesity-associated risks: A systematic review and meta-analysis. *Nutrition, Metabolism and Cardiovascular Diseases*. 2013/08/01/ 2013;23(8):699-706. doi:<https://doi.org/10.1016/j.numecd.2013.04.008>

36. Soltani S, Chitsazi MJ, Salehi-Abargouei A. The effect of dietary approaches to stop hypertension (DASH) on serum inflammatory markers: A systematic review and meta-analysis of randomized trials. *Clin Nutr*. Apr 2018;37(2):542-550. doi:10.1016/j.clnu.2017.02.018

37. Steckhan N, Hohmann CD, Kessler C, Dobos G, Michalsen A, Cramer H. Effects of different dietary approaches on inflammatory markers in patients with metabolic syndrome: A systematic review and meta-analysis. *Nutrition*. Mar 2016;32(3):338-48. doi:10.1016/j.nut.2015.09.010

38. Vilela DLS, Fonseca PG, Pinto SL, Bressan J. Influence of dietary patterns on the metabolically healthy obesity phenotype: A systematic review. *Nutrition, Metabolism and Cardiovascular Diseases*. 2021/09/22/ 2021;31(10):2779-2791. doi:<https://doi.org/10.1016/j.numecd.2021.05.007>

39. Wu P-Y, Chen K-M, Tsai W-C. The Mediterranean Dietary Pattern and Inflammation in Older Adults: A Systematic Review and Meta-analysis. *Advances in Nutrition*. 2021;12(2):363-373. doi:10.1093/advances/nmaa116

40. Yeh KL, Kautz A, Lohse B, Groth SW. Associations between Dietary Patterns and Inflammatory Markers during Pregnancy: A Systematic Review. *Nutrients*. Mar 4 2021;13(3)doi:10.3390/nu13030834

41. Chiavaroli L, Nishi SK, Khan TA, et al. Portfolio Dietary Pattern and Cardiovascular Disease: A Systematic Review and Meta-analysis of Controlled Trials. *Prog Cardiovasc Dis*. May-Jun 2018;61(1):43-53. doi:10.1016/j.pcad.2018.05.004

42. Ji J, Fotros D, Sohouli MH, Velu P, Fatahi S, Liu Y. The effect of a ketogenic diet on inflammation-related markers: a systematic review and meta-analysis of randomized controlled trials. *Nutrition Reviews*. 2025;83(1):40-58. doi:10.1093/nutrit/nuad175

43. Mayr HL, Tierney AC, Thomas CJ, Ruiz-Canela M, Radcliffe J, Itsiopoulos C. Mediterranean-type diets and inflammatory markers in patients with coronary heart disease: a systematic review and meta-analysis. *Nutrition research (New York, NY)*. 2018;50:10-24. doi:10.1016/j.nutres.2017.10.014

44. Menzel J, Jabakhanji A, Biemann R, Mai K, Abraham K, Weikert C. Systematic review and meta-analysis of the associations of vegan and vegetarian diets with inflammatory biomarkers. *Scientific Reports*. 2020/12/10 2020;10(1):21736. doi:10.1038/s41598-020-78426-8

45. Moore E, Fadel A, Lane KE. The effects of consuming a Mediterranean style diet on associated COVID-19 severity biomarkers in obese/overweight adults: A systematic review. *Nutr Health*. Dec 2022;28(4):647-667. doi:10.1177/02601060221127853

46. Nordmann AJ, Suter-Zimmermann K, Bucher HC, et al. Meta-analysis comparing Mediterranean to low-fat diets for modification of cardiovascular risk factors. *Am J Med*. Sep 2011;124(9):841-51.e2. doi:10.1016/j.amjmed.2011.04.024

47. Rondanelli M, Gasparri C, Pirola M, et al. Does the Ketogenic Diet Mediate Inflammation Markers in Obese and Overweight Adults? A Systematic Review and Meta-Analysis of Randomized Clinical Trials. *Nutrients*. Nov 22 2024;16(23)doi:10.3390/nu16234002

48. Sakhaei R, Ramezani-Jolfaie N, Mohammadi M, Salehi-Abargouei A. The healthy Nordic dietary pattern has no effect on inflammatory markers: A systematic review and meta-analysis of randomized controlled clinical trials. *Nutrition*. 2019/02/01/ 2019;58:140-148. doi:<https://doi.org/10.1016/j.nut.2018.06.020>

49. Schwingshackl L, Hoffmann G. Mediterranean dietary pattern, inflammation and endothelial function: A systematic review and meta-analysis of intervention trials. *Nutrition, Metabolism and Cardiovascular Diseases*. 2014/09/01/ 2014;24(9):929-939. doi:<https://doi.org/10.1016/j.numecd.2014.03.003>

50. Schünemann HJ, Higgins JPT, Vist GE, Glasziou P, Akl EA, Skoetz N, Guyatt GH. Chapter 14: Completing ‘Summary of findings’ tables and grading the certainty of the evidence. In: Higgins JPT, Thomas J, Chandler J, Cumpston M, Li T, Page MJ, Welch VA (editors). Cochrane Handbook for Systematic Reviews of Interventions version 6.4 (updated August 2023). Cochrane, 2023. [accessed 16 October 2023]. Available from [www.training.cochrane.org/handbook](https://d.docs.live.net/1dc7279c601258ec/PhD%20Study%202/Nutrition%20Reviews/Nutrition%20Reviews%20Revisions%202024/Fifth%20Revision/www.training.cochrane.org/handbook).
